# Critical analysis of (Quasi-)Surprise for community detection in complex networks

**DOI:** 10.1038/s41598-018-32582-0

**Published:** 2018-09-27

**Authors:** Ju Xiang, Hui-Jia Li, Zhan Bu, Zhen Wang, Mei-Hua Bao, Liang Tang, Jian-Ming Li

**Affiliations:** 1 Neuroscience Research Center & Department of Basic Medical Sciences, Changsha Medical University, Changsha, 410219 Hunan China; 2 School of Management Science and Engineering, Central University of Finance and Economics, Beijing, 100081, China; 3 Department of Anatomy, Histology and Embryology, Changsha Medical University, Changsha, 410219 Hunan China; 4 School of Information Science and Engineering, Central South University, Changsha, 410083 Hunan China; 5 Jiangsu Provincial Key Laboratory of E-Business, Nanjing University of Finance and Economics, Nanjing, 210003 China; 6 Center for OPTical IMagery Analysis and Learning (OPTIMAL), Northwestern Polytechnical University, Xi’an, 710072 Shaanxi China

## Abstract

Module or community structures widely exist in complex networks, and optimizing statistical measures is one of the most popular approaches for revealing and identifying such structures in real-world applications. In this paper, we focus on critical behaviors of (Quasi-)Surprise, a type of statistical measure of interest for community structure, accompanied by a series of comparisons with other measures. Specially, the effect of various network parameters on the measures is thoroughly investigated. The critical number of dense subgraphs in partition transition is derived, and a kind of phase diagrams is provided to display and compare the phase transitions of the measures. The effect of “potential well” for (Quasi-)Surprise is revealed, which may be difficult to get across by general greedy (agglomerative or divisive) algorithms. Finally, an extension of Quasi-Surprise is introduced for the study of multi-scale structures. Experimental results are of help for understanding the critical behaviors of (Quasi-)Surprise, and may provide useful insight for the design of effective tools for community detection.

## Introduction

Networks with complex architectures of connections are ubiquitous in nature. Biological, technological and social networks are found to exhibit many common topological properties, such as clustering, correlation and modularity^1^. The latter feature means that the networks often consist of communities, clusters or modules, i.e., groups of vertices within which connections are very dense while between which they are sparser^1^. Communities are closely related to real functional groupings in real-world systems^2,3^ and can affect dynamical behaviors such as information diffusions and synchronizations^4–6^. Because of the relevance in practical applications, many methods have been proposed to detect graph’s communities, such as spectral analysis^7,8^, random walks^9–12^, synchronization^13,14^, diffusion^15^, statistical models^16,17^, label propagation^18–21^, optimization^22–26^ and others^27–35^. Most of them consist in optimizing quality functions that can capture the intuition of community structures, such as Modularity^36^, Hamiltonians^16,37^, (Quasi-)Surprise (*S*_*q*_)^38–40^, Significance (*S*_*g*_)^41^ and “fitness” functions^42,43^.

For real applications, it becomes crucial to analyze the methods’ behavior in depth. For instance, some methods, e.g. based on modularity optimization and Bayesian inference, were shown to have phase transitions from detectable to undetectable structures, which actually constitute a limitation for their achievable performance^44–46^. On the other hand, the limits of Modularity, such as the resolution limit^47–49^, implies the possible existence of multi-scale structures in networks, and promoted the proposal of various (improved) methods, especially the multi-resolution Modularity or Potts-based Hamiltonians^50–54^. Various approaches were proposed to improve the Modularity-based methods^55,56^. For example, Lai *et al*. improved the Modularity-based belief propagation method by using the correlation between communities to improve the estimating of number of communities^56^.

Surprise (Sp) is a recently proposed statistical measure of interest for community structure, which is defined as the minus logarithm of the probability that the number of intra-community links larger or equal to the observed one is found in random networks, according to a cumulative hyper-geometric distribution^38^. While it has been shown to have good performance in many networks^38,39,57^, it is inherently applicable only to un-weighted networks and it is not easy to be optimized directly due to computational complexity caused by complex nonlinear factors. Recently, Traag *et al*.^40^ proposed a kind of accurate asymptotic approximation for Surprise (a method called Quasi-Surprise, Sq). This makes Surprise able to treat weighted networks naturally, and more accessible for theoretical analysis and efficient optimization.

So far, there is less work on the behaviors of (Quasi-)Surprise, though it has been applied to the investigation of module structures. In many networks, by comparing with Modularity as a reference, (Quasi-)Surprise seems to be immune to the resolution limit, because it has super high resolution, and thus it is able to discover the underlying community structure better. However, there may be misunderstanding needed to be clarified here. Moreover, a kind of “potential well” effect is observed in (Quasi-)Surprise, while not in Modularity and Significance. It may make (Quasi-)Surprise more difficult to be optimized than other measures such as Modularity, even if optimization for the measures remains NP-hard.

To analyze the critical behaviors of (Quasi-)Surprise in community detection, we firstly study the effect of the various network parameters on (Quasi-) Surprise, derive the critical number of dense subgraphs in merging/splitting of communities, and provide a kind of phase diagrams to display the parameter regions of dense subgraphs merging, accompanied by a series of comparisons with other methods, e.g. Modularity and Significance. And then, we will show that single group of dense subgraphs merging may be more difficult, due to the effect of “potential well” on optimization algorithms, where (Quasi-)Surprise is not a monotonic function of the number of dense subgraphs merging. This may lead to “false” optimal solutions for (Quasi-)Surprise. Moreover, we further show that the heterogeneity of degrees and community sizes will quicken the splitting of communities. Finally, we propose a kind of multi-scale version of Quasi-Surprise as well as Significance to deal with multi-scale networks.

## Results

### Effect of network parameters

**Figure 1:**
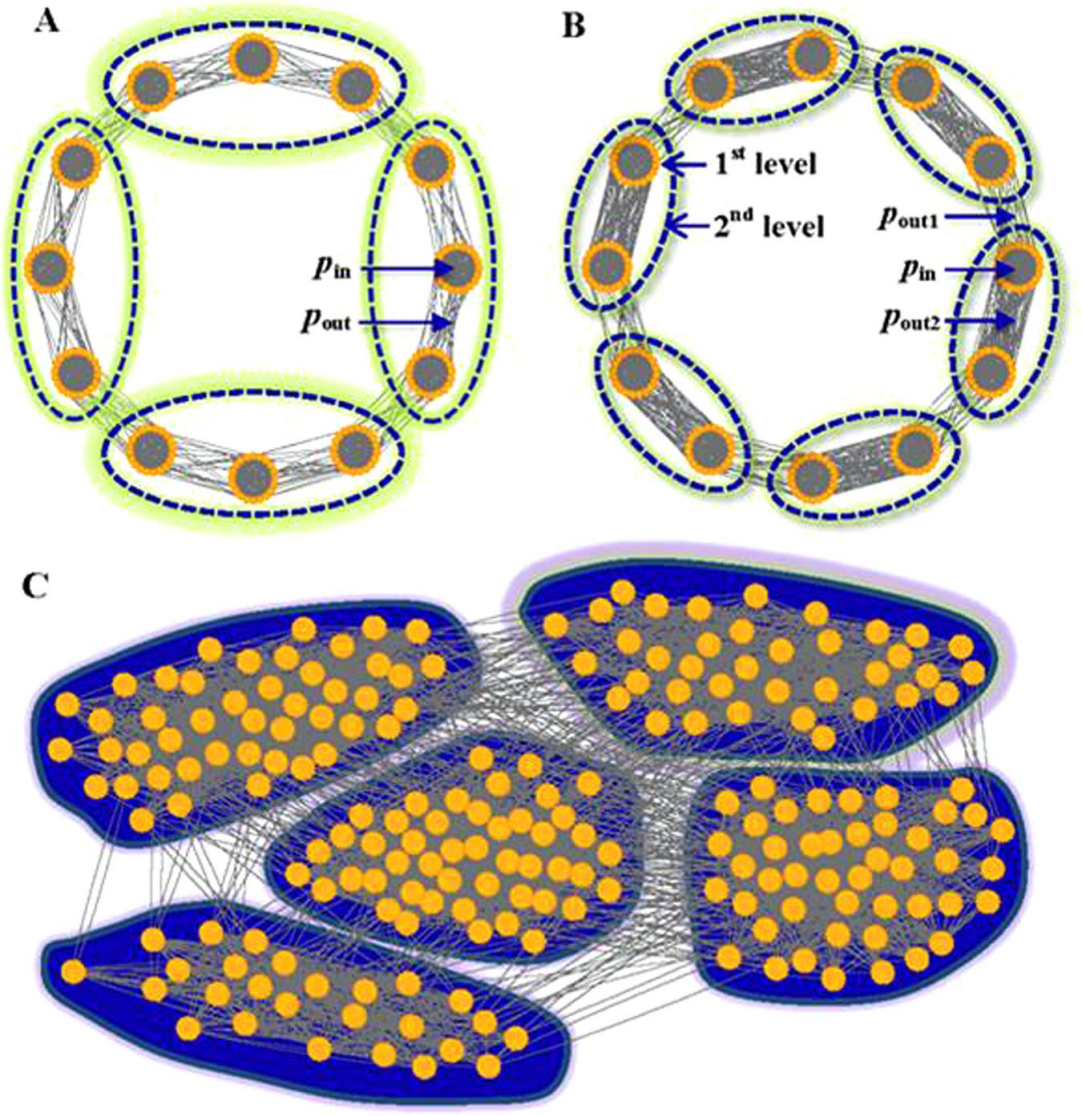
Illustration for networks. (**A**) A single-level network containing 12 dense subgraphs (i.e. 12 predefined communities), and a community partition consisting of 4 communities (Partition X = 3, marked by dash lines), where each of these communities is formed by the merging of *x* = 3 adjacent predefined communities. (**B**) Double-level networks and community structures. (**C**) LFR network and community structure.

For convenience, we have constructed a set of single-level networks with *r* “dense subgraphs” (or called “predefined communities”) that are placed at a circle and are connected to adjacent “dense subgraphs” (see Method and Fig. 1A). In those networks, let us consider a community partition that consists of *r/x* communities, where each of these communities contains *x* adjacent and dense subgraphs (or say, predefined communities), denoted by Partition X (see Fig. 1A for an example of a partition with X = 3). The pre-defined partition in the network is a special case for *x* = 1. For Partition X in the networks, the probability that a link exists within a community can be written as,

1 $$\begin{array}{rcl}{q}_{x} & = & \frac{{m}_{in}}{m}=\frac{r\cdot {n}_{c}^{2}{p}_{in}+\frac{2r}{x}(x-1){n}_{c}^{2}{p}_{out}}{r\cdot {n}_{c}^{2}{p}_{in}+2r\cdot {n}_{c}^{2}{p}_{out}}\\  & = & \frac{{p}_{in}+\frac{(x-1)}{x}2{p}_{out}}{{p}_{in}+2{p}_{out}},\end{array}$$

and the expected value of such probability can be written as,

2 $${\bar{q}}_{x}=\frac{{M}_{in}}{M}=\frac{\frac{r}{x}{(x{n}_{c})}^{2}}{{(r\cdot {n}_{c})}^{2}}=\frac{x}{r}.$$

Here, *m*_*in*_ is the number of existing intra-community links in the partition with *r/x* communities; *m* is the number of existing links in the network; n_c_ is the number of vertices in each of dense subgraphs (or say, predefined communities); *p*_*in*_ is the probability of linking vertices within the same dense subgraphs; *p*_*out*_ is the probability of linking vertices respectively in two adjacent dense subgraphs; *M*_int_ is the maximal possible number of intra-community links in the partition; *M* is the maximal possible number of links in a network. As a result, $S=mD({q}_{x}||{\bar{q}}_{x})$ for the partition, which is a multivariate function (See Methods for details).

**Figure 2:**
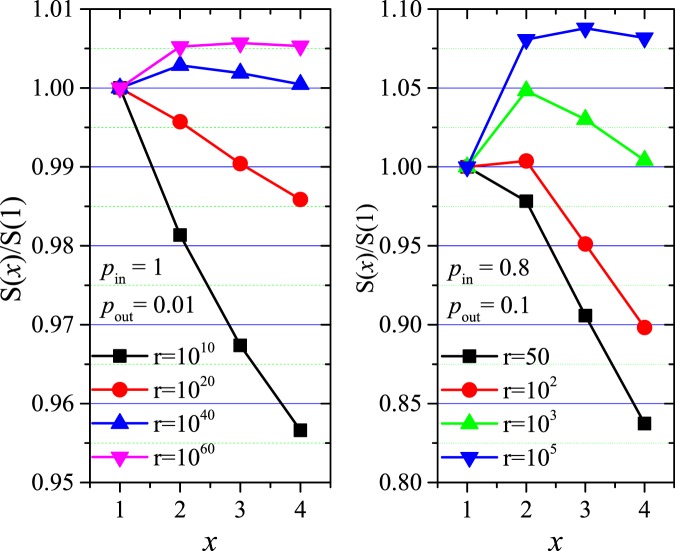
Relation between Quasi-Surprise *S*(*x*) and the number *x* of adjacent dense subgraphs of each group in distinct networks, normalized by *S*(*x* = 1), i.e., by the value of Quasi-Surprise of the pre-defined partition.

Firstly, we show the relation between Quasi-Surprise and the number *x* of dense subgraphs being contained in each community of Partition X (Fig. 2). For small *r*-values, *S*(*x*)/S(1) is less than 1 and decreases with *x*, so there is no merging of dense subgraphs; and as expected, when *r* is large enough, *S*(*x*)/S(1) has a clear peak, so the merging of *x* dense subgraphs will appear. In the most modular networks (Fig. 2, Right), where clique-like dense subgraphs are connected by only one inter-community link, the merging is very difficult or even almost impossible (because it requires large *r*-values), but, for other parameters (e.g. Fig. 2, Left), the merging is easier obviously. The difficulty of dense subgraphs merging will greatly reduce with the increase of *p*_*out*_/*p*_*in*_.

**Figure 3:**
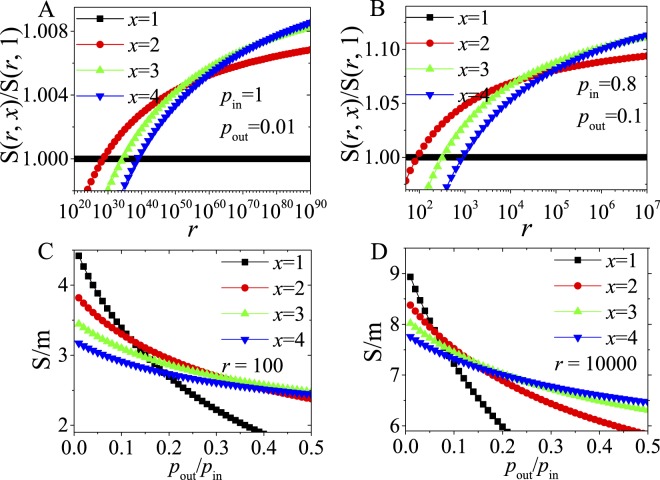
(**A**,**B**) Relation between *S* and *r* for distinct x-values, normalized by the *S*-values of the pre-defined partition. (**C**,**D**) Relation between *S* and *p*_*out*_/*p*_*in*_ for distinct x-values, normalized by the number m of edges in the networks.

Secondly, we compare the normalized *S*(*r*)*-*curves for different *x*-values (Fig. 3A,B). The *S*(*r*)-values increase with *r*. For small *r*-values, *S*(*r*, *x* = 1) is larger than others. With the increase of *r*, *S*(*r*, *x* = 2, 3 and 4) will be larger than others in turn, that is, the merging of (*x* = 2, 3 and 4) dense subgraphs will be preferred. As in Fig. 2, the merging is very difficult to appear in the most modular networks (Fig. 3A), but it may appear much more easily in other networks (e.g. Fig. 3B).

Thirdly, we show that for different *x*-values, the (normalized) *S*-curves decrease with *p*_*out*_/*p*_*in*_ (Fig. 3C,D). For small *p*_*out*_/*p*_*in*_-values, *S*(*x* = 1) is larger than others, so there is no merging. With the increase of *p*_*out*_/*p*_*in*_, *S*(*x* = 2, 3 and 4) will be larger than others in turn, that is, the (*x* = 2, 3 and 4) merging will be preferred in turn. And for larger-size networks (i.e. larger *r*-values), the merging will appear more easily.

For the sake of comparison, we also analyzed the effect of network parameters on other quality functions, the original Surprise, Significance and Modularity, which have similar behaviors to Quasi-Surprise (see Figs S1–S3).

### Phase transition in merging/disconnecting dense subgraphs

When the number *r* of dense subgraphs in the networks or *p*_*out*_/*p*_*in*_ is large enough, the dense subgraphs may merge. To study the critical number ${r}_{x}^{\ast }$ of dense subgraphs, we firstly analyze the transition of Quasi-Surprise from one partition to another in the single-scale networks (see Method). When ${\rm{\Delta }}S=S(x)-S(1)\ge 0$, the predefined partition was not preferred, compared with Partition X. By solving Δ*S* = 0 for *r*, one can obtain,

3 $${r}_{x}^{\ast }\approx \frac{1-{q}_{x}}{{q}_{x}}\exp (\frac{1}{\beta }D({q}_{1}||{q}_{x})){x}^{\frac{{q}_{1}}{\beta }+1},$$

where $\beta =\frac{x-1}{x}\frac{2{p}_{out}}{{p}_{in}+2{p}_{out}}$. For comparison, we analytically derive the critical point of Modularity for *r/x* groups of *x* dense subgraphs merging in the networks (see Method),

4 $${r}_{x}^{\ast }=\frac{{p}_{in}+2{p}_{out}}{{p}_{out}}\frac{x}{2}.$$

**Figure 4:**
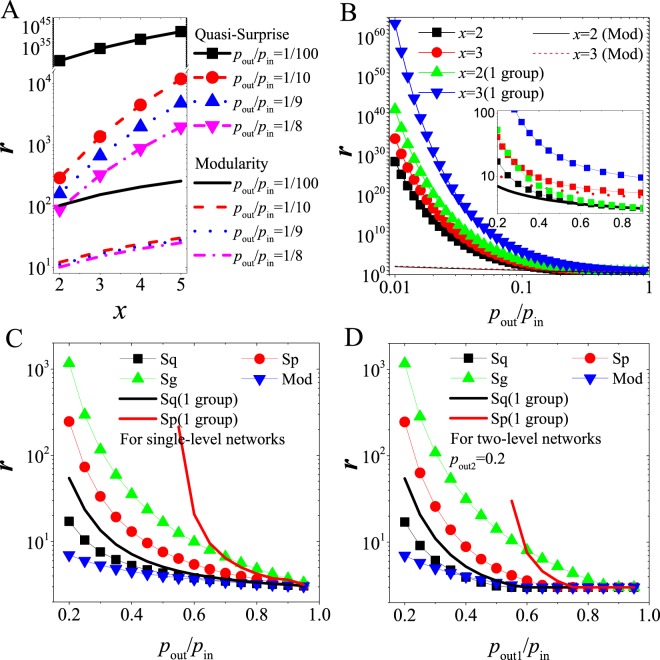
(**A**) For Quasi-Surprise and Modularity, relation between critical *r*-values and *x* for different *p*_*out*_/*p*_*in*_-values (lines + symbol for Quasi-Surprise, while the corresponding lines for Modularity) in single-level networks. (**B**) For Quasi-Surprise and Modularity, relation between critical *r*-values and *p*_*out*_/*p*_*in*_ for different *x*-values in single-level networks. The inset graph is for more explicitly comparison. (lines + symbol for Quasi-Surprise, while the corresponding lines for Modularity (Mod)). “(1 group)” denotes community partitions where there are just several (e.g. *x* = 2 or 3) “dense subgraphs” merging into 1 group, generating a community with 2 or 3 “dense subgraphs”, and other “dense subgraphs” are still considered as independent communities. (**C**) For comparison of different methods (Sq, Sp, Sg and Mod), the critical *r*-values in partition transition as a function of *p*_*out*_/*p*_*in*_ in single-level networks. (**D**) For comparison of different methods, the critical *r*-values in partition transition as a function of *p*_*out*1_/*p*_*in*_ in double-level networks.

See Fig. 4 and Supplementary information (SI) for the critical points of other measures in the networks.

For Quasi-Surprise, the critical number ${r}_{x}^{\ast }$ of dense subgraphs increases monotonically with the increase of *x* for different *p*_*out*_/*p*_*in*_-values (Fig. 4A), meaning that, the larger the *x*-values, the larger the critical ${r}_{x}^{\ast }$-values. And ${r}_{x}^{\ast }$ decays quickly with the increase of *p*_*out*_/*p*_*in*_ for all *x*–values (Fig. 4B), meaning that, the larger the *p*_*out*_/*p*_*in*_–values, the more easily the merging appears, in other words / namely, the lower the resolution of it. Moreover, the resolution of Quasi-Surprise is far higher than that of Modularity for small *p*_*out*_/*p*_*in*_–values.

Figure 4C provides a comparison of the critical *r-*values of various methods (Quasi-Surprise, Surprise, Significance and Modularity) in the single-level networks (see SI). On one hand, the critical *r*-values of the methods as a function of *p*_*out*_/*p*_*in*_ have similar behaviors; on the other hand, the curves of the critical *r*-values give a precise comparison of the resolutions of the methods.

Figure 4 could be regarded as a kind of phase diagrams in which, below the corresponding curve, the *x*-community-merging partition is not allowed compared with the pre-defined one. And the phase diagrams show that the appearance of community merging is not as difficult as imagined, e.g., in the most modular networks with small *p*_*out*_/*p*_*in*_-values. Note that, for simplicity, we have only considered the transition from the predefined partition to Partition X. Through more detailed analysis, e.g., comparing Partition X = 3 with Partition X = 2, one can find that the critical *r*-values will be larger than estimated above (see Method). Moreover, notice Fig. 4D and SI for phase transition of various methods in double-level networks.

We further verify the above conclusions directly by using Louvain algorithm to optimize the quality functions. Table 1 shows that, in the single-level networks, (a) for the fixing number *r* of dense subgraphs, dense subgraphs merging will appear when *p*_out_ is large enough for all methods, because the increase of the links between dense subgraphs makes dense subgraphs more and more close together; (b) with the increase of *r*-values, the needed *p*_out_-values for dense subgraphs merging become smaller, meaning that dense subgraphs merging become easier; (c) the effect of “potential well” appears in some cases where f* is less than 1 but there is no merging (see the “potential well” in the following section); (d) Modularity is more inclined to merge dense subgraphs than other methods, while (Quasi-)Surprise and Significance display relatively high resolution (partly due to the effect of “potential” well). (e) By comparing results of different *p*_in_-values, the decrease of *p*_in_-values will make the needed *p*_out_-values and *r*-values for dense subgraphs merging become smaller (see Tables 1 and S1), and (f) in the double-level networks, there are similar results (see Tables S2 and S3).

**Table 1:** “F1|F2|F3|F4” (the numbers separated by vertical lines) denotes the ratio of the number of identified communities by Sq, Sp, Sg and Q respectively, to that of pre-defined communities (i.e. dense subgraphs), in the single-level networks with different number of pre-defined communities and different p_out_-values (p_in_ = 1.0).

*p*_out_	4	Number of pre-defined communities		32
		8	16	
0.1	**1.00**|**1.00**|**1.00**|**1.00** (1.82|2.53|4.53|1.38)	**1.00**|**1.00**|**1.00**|**1.00** (1.27|1.66|2.24|1.05)	**1.00**|**1.00**|**1.00**|0.59 (1.13|1.40|1.69|0.96)	**1.00**|**1.00**|**1.00**|0.45 (1.07|1.28|1.45|0.93)
0.2	**1.00**|**1.00**|**1.00**|**1.00** (1.60|2.31|4.05|1.27)	**1.00**|**1.00**|**1.00**|0.53 (1.09|1.46|1.94|0.95)	**1.00**|**1.00**|**1.00**|0.43 (**0.97**|1.23|1.47|0.87)	0.64**|1.00**|**1.00**|0.33 (0.93|1.12|1.27|0.84)
0.3	**1.00**|**1.00**|**1.00**|**1.00** (1.43|2.14|3.66|1.17)	0.93|**1.00**|**1.00**|0.58 (0.94|1.27|1.67|0.86)	0.58|**1.00**|**1.00**|0.43 (0.85|1.08|1.29|0.80)	0.51**|1.00**|**1.00|**0.28 (0.82|**0.98**|1.12|0.77)
0.4	**1.00**|**1.00**|**1.00|1.00** (1.20|1.86|3.13|1.05)	0.58|**1.00**|**1.00**|0.55 (0.81|1.11|1.44|0.79)	0.50|**1.00**|**1.00|**0.41 (0.75|**0.95**|1.12|0.73)	0.44|**1.00**|0.57|0.27 (0.73|**0.88**|0.99|0.71)
0.5	0.95|**1.00**|**1.00**|0.65 (0.98|1.59|2.61|0.94)	0.58**|1.00**|**1.00**|0.40 (0.70|**0.96**|1.24|0.72)	0.46|**1.00**|0.55|0.33 (0.66|**0.83**|0.98|0.67)	0.46|**1.00**|0.54|0.24 (0.65|**0.78**|0.88|0.66)
0.6	0.50|**1.00|1.00**|0.50 (0.78|1.31|2.11|0.83)	0.53|**1.00**|**1.00**|0.45 (0.60|**0.83**|1.05|0.65)	0.45|0.83|0.55|0.31 (0.59|0.73|0.86|0.62)	0.44|0.47|0.56|0.22 (0.59|0.70|0.78|0.61)
0.7	0.50|**1.00**|**1.00**|0.55 (0.62|1.08|1.69|0.72)	0.53|0.53|0.53|0.43 (0.52|0.71|0.89|0.60)	0.45|0.56|0.56|0.33 (0.52|0.65|0.75|0.58)	0.44|0.44|0.56|0.24 (0.53|0.62|0.70|0.57)
0.8	0.50|0.60|**1.00**|0.55 (0.45|0.83|1.25|0.61)	0.43|0.60|0.58|0.45 (0.44|0.60|0.74|0.55)	0.44|0.56|0.56|0.31 (0.47|0.57|0.66|0.54)	0.43|0.44|0.44|0.22 (0.48|0.56|0.62|0.53)
0.9	0.50|0.50|0.60|0.50 (0.31|0.60|0.85|0.50)	0.50|0.55|0.55|0.43 (0.38|0.50|0.61|0.50)	0.44|0.56|0.56|0.34 (0.42|0.49|0.57|0.50)	0.36|0.44|0.45|0.22 (0.44|0.49|0.55|0.50)

“(f1|f2|f3|f4)” (the numbers in the parentheses, separated by vertical lines) denotes the ratio of the values of quality functions of pre-defined partitions for Sq, Sp, Sg and Q respectively, to that of Partition X = 2. If F1, F2, F3 or F4 is less than a value of 1, then there are the appearance of communities merging, that is, there are at least 2 or more communities being merged into a large community by corresponding methods (Sq, Sp, Sg or Q). If f1, f2, f3 or f4 is less than a value of 1, then Partition X = 2 should be preferred by Sq, Sp, Sg or Q, but the identified partitions are not inevitably to be Partition X = 2 or the partitions with communities merging, which are related to used algorithms due to the “potential well” effect.

Moreover, the above methods are applied to a set of real-word networks (Table S7). Similar to the results in the model networks, Quasi-Surprise has higher resolution than Modularity, and thus it tends to generate more communities in the real-world networks than Modularity. Original Surprise and Significance can find more communities in the networks than other methods, because they have higher resolution than others.

### Effect of “potential well” on community detection

Because of nonlinearity of (Quasi-)Surprise, a kind of unexpected phenomenon may appear when one searches the optimal community structure for (Quasi-)Surprise in an agglomerative manner, e.g., by Louvain algorithm. Generally, for some statistical measures, e.g., Modularity, one (or two) set(s) of *x*-dense subgraphs merging will be allowed, or say it can lead to the increase of the statistical measure, when Partition X is allowed, namely, it can stimulate the increase of the measure. However, it may not be the case for (Quasi-)surprise in some cases, because (Quasi-) Surprise as a function of the number of dense subgraphs merging has obvious region of low S-value, which may be difficult to get across by general greedy (agglomerative or divisive) algorithms. We call the phenomenon as “potential well” effect by borrowing the concept in Quantum mechanics.

Now, consider a partition with only one group of *x* dense subgraphs merging in the single-level networks. For small increments of *q* and $\bar{q}$, by comparing the original partition, the increments of Quasi-Surprise can be estimated by,

5 $$\begin{array}{rcl}{\rm{\Delta }}S & \approx  & \frac{\partial S}{\partial q}{\rm{\Delta }}q+\frac{\partial S}{\partial \bar{q}}{\rm{\Delta }}\bar{q}=\,\mathrm{ln}(\frac{{q}_{1}}{{\bar{q}}_{1}}\cdot \frac{1-{\bar{q}}_{1}}{1-{q}_{1}}){\rm{\Delta }}q+(\frac{1-{q}_{1}}{1-{\bar{q}}_{1}}-\frac{{q}_{1}}{{\bar{q}}_{1}}){\rm{\Delta }}\bar{q}\\  & = & \mathrm{ln}(\frac{{p}_{in}}{2{p}_{out}}\cdot r)\frac{2(x-1){p}_{out}}{{p}_{in}+2{p}_{out}}\cdot \frac{1}{r}+\frac{2{p}_{out}-r\cdot {p}_{in}}{{p}_{in}+2{p}_{out}}\cdot \frac{({x}^{2}-x)}{r},\end{array}$$

where ${\rm{\Delta }}q=\frac{2(x-1){p}_{out}}{{p}_{in}+2{p}_{out}}\cdot \frac{1}{r}$ and ${\rm{\Delta }}\bar{q}=({x}^{2}-x)/r$. By solving Δ*S* = 0 for *r*, one can obtain,

6 $${r}_{x^{\prime} }^{\ast }\approx \frac{2{p}_{out}}{{p}_{in}}\exp (\frac{{p}_{in}}{{p}_{out}}\cdot \frac{x}{2}).$$

Figure 4B clearly shows that the line “*x* = 2(1 group)” is above the line “*x* = 2”, and the line “*x* = 3(1 group)” is above the line “*x* = 3”, where “(1 group)” denotes community partitions that have only several (e.g. *x* = 2 or 3) “dense subgraphs” merging into 1 group and other “dense subgraphs” are still separated. This is a counterintuitive result, because single group of *x* dense subgraphs merging is more difficult to happen than *r/x* groups of *x* dense subgraphs merging.

**Figure 5:**
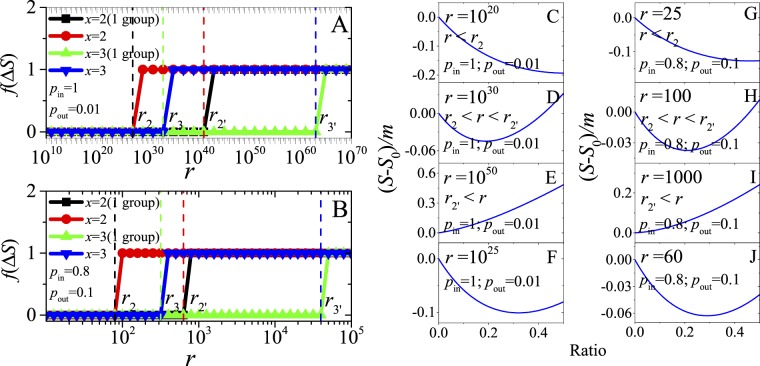
(**A**,**B**) Critical points of communities merging in the networks with different parameters. *r* is the number of dense subgraphs (i.e. predefined communities). Δ*S* denotes the increment of S. For illustration of the critical points, define the function f as *f*(Δ*S* > 0) = 1 and *f*(Δ*S* > 0) = 0. “*x* = 2(1 group)” denotes the partitions where there are only 2 dense subgraphs merging into 1 group, generating a community with 2 dense subgraphs, and other dense subgraphs are considered to be separated communities. (**C**–**J**) For Quasi-Surprise, the longitudinal coordinates are the increment of *S*, normalized by the number of edges in the networks, where *S*_0_ is the *S*-value of the original partition. And the horizontal ordinates are the ratio of the number of communities with 2 dense subgraphs to the number of dense subgraphs in the networks (denoted by Ratio).

Figure 5A,B confirms the conclusion again. When *r* > *r*_2_, the partition for *r/*2 groups of 2 dense subgraphs merging already has larger *S*-value than the predefined one. However, only when $r > {r}_{2^{\prime} }$, generating one group of 2 dense subgraphs merging is able to be approved in the agglomerative algorithms, because it will lead to the decrease of *S* before $r > {r}_{2^{\prime} }$, even if *r* > *r*_2_ (see Fig. 5C–J). This may lead that the expected 2-community-merging partition cannot be found until $r > {r}_{2^{\prime} }$.

Figure 5C–J clearly shows that, the increment of *S* (Δ*S*) decreases monotonously with the number of groups of 2 dense subgraphs merging, when *r* < *r*_2_; while it increases monotonously when $r > {r}_{2^{\prime} }$. In the two cases, generally agglomerative algorithms should be able to find the optimal partition for *S*. However, when ${r}_{2} < r < {r}_{2^{\prime} }$, there exists “potential well”, where Δ*S* will firstly decrease and then increase. It is also the case for 3-community merging. It may be difficult for general greedy algorithms to get across the “potential well”. This is frustrating, because this may lead “false” optimal results for some algorithms; while from another viewpoint, this may be a “good” news, because this means that *S* will be more difficult to encounter the resolution problem than estimated by Equation (3).

Whether could the “potential well” problem be solved by divisive algorithms? The answer may be negative. Because another kind of “potential well” still exists in some cases for the algorithms. As shown in Fig. 5F,J, *S* of Partition X = 2 is less than that of Partition X = 1, but the disconnecting of dense subgraphs or the decrease of fraction of dense subgraphs merging will lead to the decrease of *S*. Because of the existence of the “potential well”, general greedy divisive algorithms may be unable to get across the “potential well”, e.g., from Partition X = 2 to Partition X = 1. Maybe only some ergodic but time-consuming algorithms could solve the “potential well” problem to find the “true” optimal partition for Quasi-Surprise.

Does the “potential well” problem also exist in other methods? For comparison, we have confirmed that the original Surprise also has the “potential well”, while it was not found in Significance and Modularity, because of the additional property for communities (see Fig. S4). We have proved strictly that Significance have the same critical point for *r/*2 groups and single group of 2 dense subgraphs merging,

7 $${r}_{2}={r}_{2^{\prime} }=({p}_{in}+2{p}_{out})\cdot \exp (\frac{2H({p}_{2})-H({p}_{1})}{2{p}_{2}-{p}_{1}}),$$

where $H(y)=-\,y\,\mathrm{ln}(y)-(1-y)\,\mathrm{ln}(1-y)$, *p*_1_ = *p*_*i*_, *p*_2_ = (*p*_*i*_ + *p*_*o*_)/2 and *p* = (*p*_*i*_ + 2*p*_*o*_)/*r*. Modularity also has the same critical point ${r}_{2}={r}_{2^{\prime} }={p}_{in}/{p}_{out}+2$ in the two cases. Therefore they have no “potential well” effect (see SI for the proof).

**Figure 6:**
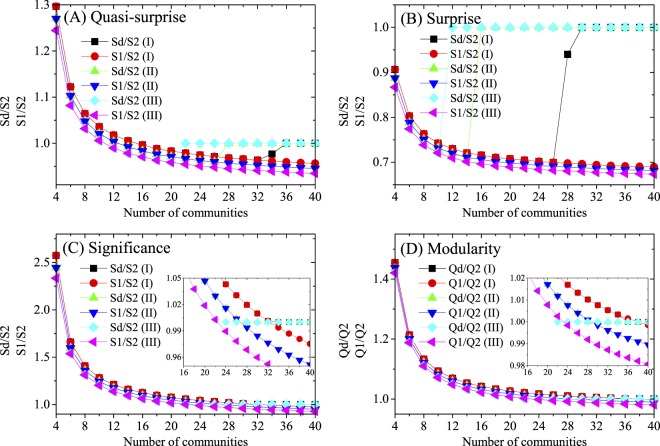
Effect of “potential” well in community detection. S1/S2, Sd/S2, Q1/Q2 and Q/Q2 as a function of the number of (the first-level) communities in the two-level networks. S1 (Q1), S2 (Q2) and Sd (Qd) denote the values of quality functions respectively for the first-level partition (without communities merging), the second-level partition (with communities merging), and the identified partition by different methods. Each measure is tested in three networks, denoted by I, II and III respectively. The parameters in the networks are set as follows: (**A**) Quasi-Surprise: *p*_i_ = 1, *p*_out1_ = 0.19(I), 0.20(II) and 0.21(III), *p*_out2_ = 0.01. (**B**) Surprise: *p*_i_ = 1, *p*_out1_ = 0.53(I), 0.54(II) and 0.55(III), *p*_out2_ = 0.1. (**C**) Significance: *p*_i_ = 1, *p*_out1_ = 0.38(I), 0.40(II) and 0.42(III), *p*_out2_ = 0.2. (**D**) Modularity: *p*_i_ = 1, *p*_out1_ = 0.03(I), 0.04(II) and 0.05(III), *p*_out2_ = 0.01.

Table 1 just shows a little sign of the “potential well” effect, therefore we have further tested the “potential well” effect of distinct measures by directly optimizing the measures (Fig. 6). As discussed above, when the number *r* of dense subgraphs is large enough, S2 (Q2) > S1 (Q1), dense subgraphs should merge. In the networks, for example, the identified partition should have a transition from the first-level partition to the second-level one. However, because of the effect of “potential” well, with the increase of *r*-values, Quasi-Surprise and Surprise have clear delays for the transition – the identified partition is still the first-level one, though S2 (Q2) > S1 (Q1). While this kind of delay is not observed for Significance and Modularity. This confirms that the effect of “potential well” really exists in Quasi-Surprise and Surprise, while not for significance and modularity.

### Effect of heterogeneity of vertex degree and community size

To study the effect of heterogeneity of vertex degrees and community sizes on Quasi-Surprise, we further apply various methods to a type of classical modular network, Lancichinetti-Fortunato- Rachicchi (LFR) networks^58^ (Fig. 1C), which are able to mimic the general properties of many real-world networks, such as the community structures, the heterogeneity of vertex degrees and community sizes.

Firstly, a set of networks with different heterogeneity of vertex degrees and community sizes are generated by the increase of maximal degree and maximal community size. The heterogeneity of the two kinds make the inhomogeneity of link density within communities emerges gradually due to the random fluctuations of links. When the inhomogeneity of link density is large enough in a community, the community may be split. The results (Tables 2 and S4 and Fig. S7) show that, (1) almost all methods work well in homogeneous networks; (2) the heterogeneity of community size leads the splitting of communities (see *Sq, Sp, Sg* and *Mod*); (3) while the heterogeneity of degree aggravates the splitting of communities further. (4) The partitions by *Sq, Sp* and *Sg* contain more groups of vertices than the pre-defined ones, because they (as well as LP^19^) are more inclined to split the communities, especially when comparing with other methods (such as Modularity^22,36^, Infomap^10^, Walktrap^11^ and OSLOM^59^).

**Table 2:** Ratio of the number of communities identified by different methods, to that of the predefined ones, in the LFR networks with different network size (N), different heterogeneity of vertex degrees and community sizes.

N	*k*_*max*_	*C*_*max*_	Sq	Sp	Sg	Mod	Infomap	Infomod	Walktrap	OSLOM	LP
200	10	10	1.00	1.00	1.00	1.00	1.00	1.00	1.00	1.00	1.11
		80	1.41	1.74	5.13	1.02	1.00	1.00	1.00	1.00	1.11
		100	1.73	2.06	5.69	1.00	1.00	1.00	1.00	0.98	1.11
	50	80	2.90	3.63	8.85	1.00	1.00	1.00	1.00	1.00	2.42
		100	3.38	4.68	10.06	1.05	1.06	1.00	1.00	1.00	3.19
500	10	10	1.00	1.00	1.00	1.00	1.00	0.68	1.00	1.00	1.11
		80	1.00	1.00	2.44	1.00	1.00	1.00	1.00	1.00	1.11
		100	1.00	1.02	3.43	1.00	1.00	1.00	1.00	1.00	1.11
	50	80	1.35	1.71	5.32	1.00	1.00	0.94	1.00	1.00	2.82
		100	2.19	2.54	6.78	1.00	1.03	0.93	1.00	1.00	2.30

Other parameters: *k*_m_ = 10, *C*_min_ = 20, *µ* = 0.1, τ_1_ = 2, and τ_2_ = 2. The increase of *k*_*max*_ and *C*_*max*_ will respectively lead the increase of the heterogeneity of degree and community size in the networks.

Then, we study the effect of the mean degree of the networks. With the decrease of the mean degree of the networks, links within communities become more and more sparse. Thus, the inhomogeneity of link density will emerge gradually in the communities due to the random fluctuations. As a result, communities will also tend to split (see Tables S5 and S6, Figs S8–S11 and the details of analysis).

Further, we also analyzed the effect of the network size on the above results. For Quasi-Surprise, Surprise, Significance and Modularity, with the increase of network size, (1) the tendency to split is also weakened gradually; (2) the difference between identified and pre-defined partition gradually decreases; and (3) NMI gradually increases (see Tables 2 and S4–S6, Figs S8–S11).

### Extension to multi-scale networks

#### Analysis of critical behaviors in multi-scale networks

We further study the critical behaviors of various measures in the networks with two-scale community structures, a generalization of the single-scale networks (Fig. 1B). In the networks, the critical *r*-value of *Sq* can be estimated by (see SI for the proof),

8 $${r}_{2}^{\ast }\approx \frac{{p}_{out2}}{{p}_{in}+{p}_{out1}}\cdot \exp (\frac{{p}_{in}}{{p}_{out1}}\,\mathrm{ln}\,\frac{{p}_{in}}{{p}_{in}+{p}_{out1}}+\frac{{p}_{out1}+{p}_{out2}}{{p}_{out1}}\,\mathrm{ln}\,\frac{{p}_{out1}+{p}_{out2}}{{p}_{out2}})\cdot {2}^{\frac{{p}_{in}}{{p}_{out1}}+1}.$$

For only one group of 2 (first-level) communities merging in the networks,

9 $${r}_{2^{\prime} }^{\ast }\approx \frac{{p}_{out1}+{p}_{out2}}{{p}_{in}}\exp (\frac{{p}_{in}}{{p}_{out1}}).$$

When *p*_*out*1_ = *p*_*out*2_, the networks are equivalent to the single-scale networks. When *p*_*out*1_ > *p*_*out*2_, two-scale structures emerge. With the decrease of *p*_*out*2_/*p*_*in*_, ${r}_{2}^{\ast }$ decreases. When *p*_*out*2_ = 0, ${r}_{2}^{\ast }\approx \frac{{p}_{out1}}{{p}_{in}}\cdot {(\frac{2{p}_{in}}{{p}_{in}+{p}_{out1}})}^{\frac{{p}_{in}}{{p}_{out1}}+1}$, so the effect of *p*_*out*2_ is limited. ${r}_{2}^{\ast }$-value is mainly determined by *p*_*out*1_/*p*_*in*_; also, equation (9) has similar behaviors (see Fig. S6). See SI for detailed analysis of critical behaviors of various measures in the networks. We provided a systematical comparison of phase diagrams of various measures (Sq, Sp, Sg and Mod) in the networks (See Figs 4D, S5 and S6).

In the networks, (Quasi-)Surprise also has ${r}_{2} < {r}_{2^{\prime} }$ (Figs 4D, S5 and S6). On the one hand, this means the “potential well” effect still exists when ${r}_{2} < r < {r}_{2^{\prime} }$, and thus general greedy optimization algorithm may prefer either the first-level partition (for agglomerative algorithms, see Fig. 6) or the second-level partition (for divisive algorithms), leading to “false” optimal solutions. So the identified level depends on applied algorithms.

On the other hand, the partition of which level is identified is closely related to the number of communities in the networks, partly because (Quasi-)Surprise is just single-scale method. When *r* < *r*_2_, the first level is found; when ${r}_{2^{\prime} } < r$, the second level is preferred; when ${r}_{2} < r < {r}_{2^{\prime} }$, the identified level depends on whether optimization algorithm could get across the “potential well” to find true optimal solutions. Moreover, the critical values of ${r}_{2^{\prime} }$ (as well as *r*_2_) will quickly decrease with the increase of *p*_*out*1_/*p*_*in*_, and therefore small *p*_*out*1_/*p*_*in*_-values will prefer the first level in a network with fixed *r*, while large *p*_*out*1_/*p*_*in*_-values will prefer the second level (in this case, the communities easily merge).

Because of the accumulative property, Modularity has the same critical ${r}_{2}^{\ast }$-value for *r/*2 groups and single group of 2 communities merging in the networks: ${r}_{2}^{\ast }=({p}_{in}+{p}_{out1}+{p}_{out2})/{p}_{out1}$, and Significance is too: ${r}_{2}^{\ast }=({p}_{in}+{p}_{out1}+{p}_{out2})\cdot \exp \{(2H({p}_{2})-H({p}_{1}))/(2{p}_{2}-{p}_{1})\}$, where *p*_1_ = *p*_*in*_ and *p*_2_ = (*p*_*in*_ + *p*_*out*1_)/2 (see SI). They thus do not show the potential-well phenomenon, but there still is similar resolution problem in the networks: the second level is found when $r > {r}_{2}^{\ast }$, otherwise the first level is found.

#### Extension to multi-scale community detection

As discussed above, (Quasi-) Surprise as well as many other methods are just single-scale methods with limited resolutions, so the identified partitions or levels closely depend on the network parameters such as the (inter- and intra-community) link densities and the number of communities in the networks (note that the “potential well” effect is also related to the number of communities). And multi-scale structures extensively exist in various complex networks. So, developing multi-resolution (or multi-scale) methods is of importance. Here, we proposed an extension of Quasi-Surprise to multi-scale networks, by adjusting the random model (See Methods;), because the original Quasi-Surprise closely depends on the difference between the probability of links existing within communities and the expected values in the random model. Similarly, other measures such as Significance and Modularity can also be extended to multi-scale networks (see Methods). The exact formulations of the methods are provided in the additional material.

**Figure 7:**
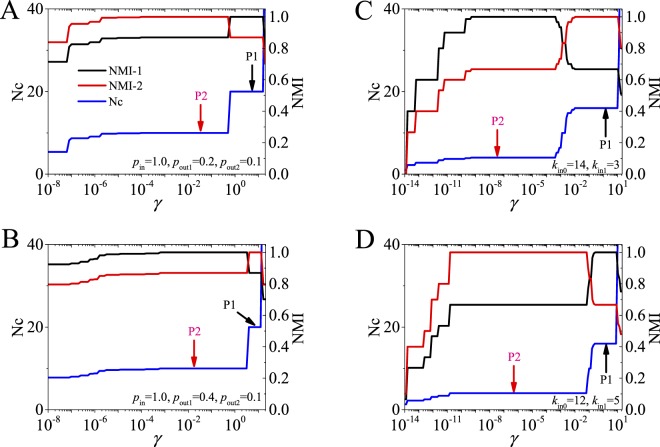
Community partitions identified by Quasi-Surprise in (**A**,**B**) double-level networks with *n*_c_ = 10 and *r* = 20, and (**C**,**D**) hierarchical networks with 256 vertices and two-scale community structures. Nc is the number of identified communities in the networks. NMI-1 denotes the NMI between identified and level-1 partitions. NMI-2 denotes the NMI between identified and level-2 partitions.

To display the effectiveness of the multi-scale methods in detecting communities at different scales, the methods are applied to two kinds of networks with multi-scale community structures (Figs 7, S12 and S13). The results show that they are able to identify the partitions of the pre-defined scales in the networks. The proposed multi-scale extensions are simple while effective, which provide alternative approaches to analyze the community structures at different scales.

## Discussion and Conclusion

Community structure (or module structure) widely exists in various complex networks. Detecting the communities (or modules) in complex networks is an issue of interest in the research of complex networks. Many methods have been proposed to detect community structures in complex networks, and optimizing quality functions for community structure is one of the most important strategies for community detection. The existing methods could help in discovering the intrinsic structures in networks, but they also have respective scopes of application. Therefore, it is necessary to study the behaviors of the methods for the theoretical research and real applications. This is of help in understanding the methods themselves, and can promote the improvement of the methods or the development of more effective methods. However there is less work on the behaviors of (quasi-)Surprise–a kind of statistical measure of interest for community structure until now.

This paper provided the detailed study for the critical behaviors of (Quasi-)Surprise, accompanied by a series of comparison with other methods, including Significance, Modularity, Infomap, Walktrap, LP and OSLOM. We analyzed the effect of various network parameters on (Quasi-)Surprise, and derive the critical number of dense subgraphs in merging/splitting of dense subgraphs. To display the phase transitions of various measures from one partition to another one, we provided the phase diagrams of the critical points in merging/splitting of dense subgraphs, which give a clear comparison for the critical points of various measures. The critical number of dense subgraphs for (Quasi-)Surprise has a clearly super-exponential increase with the difference between inter- and intra-community link possibilities, but it is close to that of Modularity for small difference of link possibilities, for which the difference between the resolutions of (Quasi-)Surprise and Modularity is far less than in the most modular structures.

A kind of “potential well” effect for (Quasi-) Surprise was revealed in community detection. In some cases, just one group of *x* dense subgraphs merging may be more difficult to appear than all *r/x* groups of *x* dense subgraphs merging, because, when ${r}_{2} < r < {r}_{2^{\prime} }$, (Quasi-) Surprise as a function of the number of dense subgraphs merging has obvious “potential well”. The “potential well” is generally difficult to get across by greedy (agglomerative or divisive) algorithms, e.g. the popular Louvain. This may result in “false” optimal solutions for the algorithms, though this also may be able to implicitly mitigate the resolution problem or the excessive split of communities, to some extent. Maybe, only some ergodic but time-consuming algorithms, e.g., simulated annealing, can avoid the problem.

Overall, (Quasi-) Surprise tends to split communities due to such reasons as the heterogeneity of link density, degree and community size, often displays higher resolution, and thus identify more communities than other methods, e.g., Modularity, in community detection, but it also may lead to the excessive splitting of communities due to the density inhomogeneity inside communities, e.g., caused by the heterogeneity of degrees and community sizes.

Moreover, it is believed that multi-scale structures widely exist in various complex networks. In the multi-scale networks, e.g. the double-level networks above, different methods may identify structures at different scales. (Quasi-) Surprise is a kind of statistical measures of interest for community detection, but it is just a single-scale method. And the results suggest the necessity of developing multi-resolution methods, though it may be not easy for (Quasi-)Surprise. We proposed an extension of Quasi-Surprise to multi-scale networks, which provide alternative approaches for identifying the multi-scale structures.

Finally, we expected that the above analysis could be helpful for the understanding of the critical behaviors of the statistical measures and provide useful insight for developing more effective community-detection methods in complex networks.

## Method

### Networks

#### Single-level networks

For convenience of analysis, a set of community-loop networks with single-level community structure is constructed, where *r* “dense subgraphs” (predefined communities) are placed at a circle and are connected to adjacent ones. For each network, let n_c_ the number of vertices in each (predefined) community, while *n* = *r* · *n*_*c*_ the number of vertices in the network; *p*_*in*_ is the probability of linking vertices within the same (predefined) community; *p*_*out*_ is the probability of linking vertices respectively in two adjacent (predefined) communities; $m=r\cdot {n}_{c}^{2}{p}_{in}+2r\cdot {n}_{c}^{2}{p}_{out}$ is the number of edges in the networks (Fig. 1A).

#### Double-level networks

To further analyze the phenomena in the merge and breakup of communities, we construct the community-loop networks with double-level community structures. Let *r* the number of communities and *n*_c_ the number of vertices in each community at the first level, while *n* = *r* · *n*_*c*_ the number of vertices in the network. *p*_*in*_ the probability of linking vertices within the first-level community; *p*_*out*1_ the probability of linking vertices respectively in the first-level and adjacent communities contained in the same second-level community; *p*_*out*2_(<*p*_*out*1_) the probability of linking vertices respectively in two adjacent and first-level communities contained in two different second-level communities (Fig. 1B).

#### LFR networks

Lancichinetti-Fortunato-Rachicchi (LFR) networks is a type of classical modular networks, which are able to mimic the general properties of many real-world networks, such as the community structures, the heterogeneity of vertex degrees and community sizes^58^. In the networks, vertex degrees and community sizes follow power-law distributions with exponents τ_1_ and τ_2_ respectively, and a common mixing parameter *μ* controls the ratio between the external degree of each vertex with respect to its community and the total degree of the vertex. The smaller the *μ-*values, the more obvious the communities. Here, low *μ-*values are used, so communities are well separated from each other (Fig. 1C).

#### Hierarchical networks

The networks have 256 vertices and two-scale community structures^14^. The first scale consists of 4 groups of 64 vertices and the second scale consists of 16 groups of 16 vertices. The number of links of each vertex with the most internal community is *k*_in0_, the number of links of each vertex with the most external community is *k*_in1_, and the number of links with any other vertex at random in the network is 1.

### Statistical measures for community structure

#### Surprise (*S*_*p*_)

*Surprise* is a statistical approach to assess the quality of community partition in a network, with higher values corresponding to better partitions^38^. It was shown that *Surprise* can give better characterization for community structures than modularity in several complex benchmarks. Given a community partition in a network, *Surprise* is defined as the minus logarithm of the probability that the observed number of intra-community links or more appears in Erdös-Rényi random networks. According to a cumulative hyper-geometric distribution, it can be written as,

10 $${S}_{p}=-\,\mathrm{log}\,\sum _{j={m}_{{\rm{int}}}}^{{\rm{\min }}(m,{M}_{{\rm{int}}})}\frac{(\begin{array}{c}{M}_{\mathrm{int}}\\ j\end{array})(\begin{array}{c}M-{M}_{{\rm{int}}}\\ m-j\end{array})}{(\begin{array}{c}M\\ m\end{array})},$$

where *M* is the maximal possible number of links in a network; *M*_int_ is the maximal possible number of intra-community links in a given partition; *m* is the number of existing links in the network; while *m*_int_ is the number of existing intra-community links in the partition.

#### Quasi-Surprise (*S*_*q*_)

The original definition of *Surprise* is for un-weighted networks and it involves complex nonlinear factors, leading to the difficulties of the theoretical analysis and numerical computations. So it is very useful to provide a kind of effective approximate expression for *Surprise*. By only taking into account the dominant term and using Stirling’s approximation of the binomial coefficient, a kind of Quasi-Surprise reads,

11 $$\begin{array}{rcl}{S}_{q} & \approx  & -\mathrm{log}\,\frac{(\begin{array}{c}{M}_{{\rm{int}}}\\ {m}_{{\rm{int}}}\end{array})(\begin{array}{c}M-{M}_{{\rm{int}}}\\ m-{m}_{{\rm{int}}}\end{array})}{(\begin{array}{c}M\\ m\end{array})}\\  & \approx  & m(q\,\mathrm{log}\,\frac{q}{\bar{q}}+(1-q)\,\mathrm{log}\,\frac{1-q}{1-\bar{q}})\\  & = & mD(q||\bar{q}),\end{array}$$

where *q* = *m*_int_/*m* denotes the probability that a link exists within a community; $\bar{q}={M}_{{\rm{int}}}/M$ denote the expected value of *q*; $D(x||y)=x\,\mathrm{ln}\,\tfrac{x}{y}+(1-x)\mathrm{ln}\,\tfrac{1-x}{1-y}$ is the Kullback-Leibler divergence, which measures the distance between two probability distributions *x* and *y*^40^.

The original Quasi-Surprise is based on the difference between the probability of links existing within communities and the expected values in random model. We propose a kind of alternative approach to extend the Quasi-Surprise to multi-scale case, by using a resolution parameter to adjust the expected values in random model. This results in the multi-scale Quasi-Surprise,

12 $${S}_{q}(\gamma )=mD(q||\gamma \cdot \bar{q}),$$

where *γ* is the resolution parameter.

#### Significance (*S*_*g*_)

Significance also is a recently proposed measure for estimating the quality of community structures, which looks at how likely dense communities appear in random networks^41^. It is defined as

13 $${S}_{g}=\sum _{s}(\begin{array}{c}{n}_{s}\\ 2\end{array})D({p}_{s}||p),$$

Here the sum runs over all communities; the density of community *s*, *p*_*s*_, is the ratio of the number of existing edges to the maximum in the community; the density of network, *p*, is the ratio of the number of existing edges to the maximum in the whole network. It cloud also be directly optimized as objective function to find the optimal community partitions.

To extend the original Significance to multi-scale cases, we use a parameter to adjust the density of network, because the original Significance is based on the difference between the density of community and the density of network. As a result, the multi-scale Significance can be written as,

14 $${S}_{g}=\sum _{s}(\begin{array}{c}{n}_{s}\\ 2\end{array})D({p}_{s}||\gamma \cdot p),$$

where *γ* is the resolution parameter.

#### Modularity (*Q* or Mod)

For given community division of a network, it is defined as^36^

15 $$Q=\sum _{s}\frac{{k}_{s}^{in}}{2M}-{(\frac{{k}_{s}}{2M})}^{2},$$

where *M* is the total number of edges in the network, ${k}_{s}^{in}$ the inner degree of group *s*, *k*_*s*_ the total degree of group *s*, and the sum runs over all communities in the given network. Modularity evaluates the fraction of edges within communities in the network minus the expected value in a random graph (i.e. in the null model). In general, the larger the modularity, the better the division. In recent years, it has become one of the most popular quality functions for community detection.

To detect communities at different scales, the multi-scale Modularity can be defined as,

16 $$Q=\sum _{s}\frac{{k}_{s}^{in}}{2M}-\gamma {(\frac{{k}_{s}}{2M})}^{2},$$

where *γ* is the resolution parameter.

#### Normalized mutual information (NMI)

This measure is taken from information theory and estimates the similarity between two community partitions^42^. When perfectly matched, NMI = 1. Otherwise, the less is the match, the smaller is the value of NMI. NMI is often used to evaluate the performance of methods by assessing the amount of community information correctly extracted by the methods in networks with known community structures.

### Critical number of dense subgraphs in partition transition

For the single-level networks, we derive the critical number ${r}_{{x}_{1}\to {x}_{2}}^{\ast }$ of dense subgraphs from Partition X_1_ to Partition X_2_ (Partition X denotes the partition with *r/x* groups of *x* dense subgraphs merging). When ${\rm{\Delta }}S=S({x}_{2})-S({x}_{1}) > 0$, Partition X_2_ will be preferred, compared to Partition X_1_. Here, Δ*S*, divided by the number of links, can be written as,

17 $$\begin{array}{rcl}{\rm{\Delta }}S & = & \frac{S({x}_{2})-S({x}_{1})}{m}\\  & = & {q}_{{x}_{1}}\,\mathrm{ln}(\frac{{q}_{{x}_{2}}}{{\bar{q}}_{{x}_{2}}}\frac{{\bar{q}}_{{x}_{1}}}{{q}_{{x}_{1}}})+(1-{q}_{{x}_{1}})\,\mathrm{ln}(\frac{1-{q}_{{x}_{2}}}{1-{\bar{q}}_{{x}_{2}}}\frac{1-{\bar{q}}_{{x}_{1}}}{1-{q}_{{x}_{1}}})+\beta \,\mathrm{ln}(\frac{{q}_{{x}_{2}}}{{\bar{q}}_{{x}_{2}}}\frac{1-{\bar{q}}_{{x}_{2}}}{1-{q}_{{x}_{2}}})\\  & = & {q}_{{x}_{1}}\,\mathrm{ln}(\frac{{q}_{{x}_{2}}}{{q}_{{x}_{1}}}\frac{{x}_{1}}{{x}_{2}})+(1-{q}_{{x}_{1}})\,\mathrm{ln}(\frac{1-{q}_{{x}_{2}}}{1-{q}_{{x}_{1}}}\frac{1-\frac{{x}_{1}}{r}}{1-\frac{{x}_{2}}{r}})+\beta \,\mathrm{ln}(\frac{{q}_{{x}_{2}}}{1-{q}_{{x}_{2}}}\frac{1-\frac{{x}_{2}}{r}}{\frac{{x}_{2}}{r}})\\  & \approx  & {q}_{{x}_{1}}\,\mathrm{ln}(\frac{{q}_{{x}_{2}}}{{q}_{{x}_{1}}}\frac{{x}_{1}}{{x}_{2}})+(1-{q}_{{x}_{1}})\,\mathrm{ln}(\frac{1-{q}_{{x}_{2}}}{1-{q}_{{x}_{1}}})+\beta \,\mathrm{ln}(\frac{{q}_{{x}_{2}}}{1-{q}_{{x}_{2}}}\frac{r}{{x}_{2}})\\  & \approx  & -D({q}_{{x}_{1}}||{q}_{{x}_{2}})+{q}_{{x}_{1}}\,\mathrm{ln}(\frac{{x}_{1}}{{x}_{2}})+\beta \,\mathrm{ln}(\frac{{q}_{{x}_{2}}}{1-{q}_{{x}_{2}}}\frac{r}{{x}_{2}}),\end{array}$$

where $$r-{x}_{1}\approx r-{x}_{2}\approx r$$, $$D(x||y)=x\,\mathrm{ln}\,\tfrac{x}{y}+(1-x)\,\mathrm{ln}\,\tfrac{1-x}{1-y}$$ and $$\beta =(\frac{{x}_{2}-1}{{x}_{2}}-\frac{{x}_{1}-1}{{x}_{1}})\frac{2{p}_{out}}{{p}_{in}+2{p}_{out}}$$.

By solving Δ*S* = 0 for *r*, one can obtain the critical number of dense subgraphs,

18 $$\begin{array}{rcl}{r}_{{x}_{1}\to {x}_{2}}^{\ast } & \approx  & \frac{1-{q}_{{x}_{2}}}{{q}_{{x}_{2}}}\exp (\frac{1}{\beta }D({q}_{{x}_{1}}||{q}_{{x}_{2}})){(\frac{{x}_{2}}{{x}_{1}})}^{\frac{{q}_{{x}_{1}}}{\beta }}\cdot {x}_{2}\\  & = & \frac{\frac{2{p}_{out}}{{x}_{2}}}{{p}_{in}+2{p}_{out}\frac{({x}_{2}-1)}{{x}_{2}}}\exp \{\frac{{p}_{in}+2{p}_{out}\frac{({x}_{1}-1)}{{x}_{1}}}{2{p}_{out}(\frac{{x}_{2}-1}{{x}_{2}}-\frac{{x}_{1}-1}{{x}_{1}})}\,\mathrm{ln}\,\frac{{p}_{in}+2{p}_{out}\frac{({x}_{1}-1)}{{x}_{1}}}{{p}_{in}+2{p}_{out}\frac{({x}_{2}-1)}{{x}_{2}}}\\  &  & +\frac{1}{{x}_{1}(\frac{{x}_{2}-1}{{x}_{2}}-\frac{{x}_{1}-1}{{x}_{1}})}\,\mathrm{ln}\,\frac{{x}_{2}}{{x}_{1}}\}{(\frac{{x}_{2}}{{x}_{1}})}^{\frac{{p}_{in}+2{p}_{out}\frac{({x}_{1}-1)}{{x}_{1}}}{2{p}_{out}(\frac{{x}_{2}-1}{{x}_{2}}-\frac{{x}_{1}-1}{{x}_{1}})}}\cdot {x}_{2}\end{array}$$

For comparison, in the same networks, we derive the critical point of modularity for *r/x* groups of *x* dense subgraphs merging. For Partition X,

19 $$\begin{array}{rcl}{Q}_{x} & = & \frac{r}{x}(\frac{x{p}_{in}+2(x-1){p}_{out}}{r\cdot {p}_{in}+2r\cdot {p}_{out})}-{(\frac{x}{r})}^{2})\\  & = & \frac{{p}_{in}+2\frac{x-1}{x}{p}_{out}}{{p}_{in}+2{p}_{out}}-\frac{x}{r}.\end{array}$$

By solving *Q*_*x*2_ − *Q*_*x*1_ = 0 fo*r r*, one can obtain,

20 $${r}_{{x}_{1}\to {x}_{2}}^{\ast }=\frac{{p}_{in}+2{p}_{out}}{2{p}_{out}}({x}_{2}-{x}_{1}){(\frac{{x}_{2}-1}{{x}_{2}}-\frac{{x}_{1}-1}{{x}_{1}})}^{-1}.$$

For *x*_1_ = 1, $${r}_{x}^{\ast }=\frac{{p}_{in}+2{p}_{out}}{2{p}_{out}}{x}_{2}$$.

## Electronic supplementary material

Supplementary Information
