# Supplementary material for: Critical analysis of (Quasi-)Surprise for community detection in complex networks
Source: Sci Rep. 2018 Sep 27;8:14459. doi: 10.1038/s41598-018-32582-0 (PMC6160439; doi:10.1038/s41598-018-32582-0)
Supplement: Supplementary file 1 — Supplementary Information [file 41598_2018_32582_MOESM1_ESM.doc]

**Supplementary information**

**Critical analysis of (Quasi-)Surprise for community detection in complex networks**

Ju Xiang[^1^](#First)^,3,4^, Hui-Jia Li^2^*****, Zhan Bu^5^, Zhen Wang^6^, Mei-Hua Bao[^1^](#First)^,3,4^, Liang Tang[^1^](#First)^,3,4^ &Jian-Ming Li[^1^](#First)^,3,4^*****

^1^Neuroscience Research Center, Changsha Medical University, Changsha, 410219, Hunan, China

^2^School of Management Science and Engineering, Central University of Finance and Economics, Beijing 100080, China

^3^Department of Anatomy, Histology and Embryology, Changsha Medical University, Changsha, 410219, Hunan, China

^4^Department of Basic Medical Sciences, Changsha Medical University, Changsha, 410219, Hunan, China

^5^Jiangsu Provincial Key Laboratory of E-Business, Nanjing University of Finance and Economics, Nanjing 210003, China.

^6^Center for OPTical IMagery Analysis and Learning (OPTIMAL), Northwestern Polytechnical University, Xi’an 710072, Shaanxi, China

*Correspondence and requests for materials should be addressed to Hui-Jia Li and Jian-Ming Li.

Email: [hjli@amss.ac.cn](mailto:hjli@amss.ac.cn(H.J.L.)) (HJL); [ljming0901@sina.com](mailto:ljming0901@sina.com) (JML).

Content

[**1. Effect of network parameters on original Surprise, Significance and Modularity** 4](#__RefHeading___Toc505456257)

[**2. Critical behaviors of Quasi-Surprise, Modularity, Significance and original Surprise on single-level networks** 5](#__RefHeading___Toc505456258)

[2.1 Critical number of dense subgraphs for Quasi-Surprise 5](#__RefHeading___Toc505456259)

[2.2 Critical number of dense subgraphs for Modularity 6](#__RefHeading___Toc505456260)

[2.3 Critical number of dense subgraphs for Significance 7](#__RefHeading___Toc505456261)

[2.4 Critical number of dense subgraphs for original Surprise 7](#__RefHeading___Toc505456262)

[**3. Test of “potential well” effect for original Surprise and Significance** 7](#__RefHeading___Toc505456263)

[**4. Critical behaviors of Quasi-Surprise, Modularity, Significance and original Surprise on networks with double-level community structures** 8](#__RefHeading___Toc505456264)

[4.1 Critical number of dense subgraphs for Quasi-Surprise 8](#__RefHeading___Toc505456265)

[4.2 Critical number of dense subgraphs for Modularity 9](#__RefHeading___Toc505456266)

[4.3 Critical number of dense subgraphs for Significance 10](#__RefHeading___Toc505456267)

[4.4 Critical number of dense subgraphs for original Surprise 11](#__RefHeading___Toc505456268)

[**5. Experimental results based on direct optimization** 13](#__RefHeading___Toc505456269)

[5.1 Test in community-loop networks with single- and two-level community structures 13](#__RefHeading___Toc505456270)

[5.2 Test in LFR networks: Effect of heterogeneity of degree and community size 14](#__RefHeading___Toc505456271)

[**6. Analysis of multi-scale methods** 21](#__RefHeading___Toc505456272)

[**7. Real-world networks**  23](#__RefHeading___Toc505456273)

[References 23](#__RefHeading___Toc505456274)

**All Figures**

[**Figure S1. Relation between *S* and *x* in distinct networks with *p*_in_=1 and *p*_out_=0.25, normalized by the *S*-value of the pre-defined partition (*x*=1), for (Left) original Surprise and (Right) Significance.** 4](#__RefHeading___Toc484075692)

[**Figure S2. Relation between *S* and *r* for distinct *x*-values, normalized by the *S*-values of the pre-defined partition, for (Top) original Surprise and (Bottom) Significance.** 4](#__RefHeading___Toc484075693)

[**Figure S3. Relation between *S* and** $p_{out}\text{/}p_{in}$ **for distinct *x*-values, normalized by the number of edges in the networks, for (Top) original *Surprise* and (Bottom) *Significance*.** 5](#__RefHeading___Toc484075694)

[**Figure S4. The increase of *S*,** $(S-S_{0})/m$**, as a function of the fraction of generating 2-community groups in networks with different number of dense subgraphs.** $S_{0}$ **is the *S*-value of the original partition. *p*_in_=1, *p*_out_=0.25. (a)-(c) for *Surprise*. (d)-(f) for *Significance*.** 7](#__RefHeading___Toc484075695)

[**Figure S5. In the two-level networks, effect of** $p_{out1}/p_{in}$ **on the critical number of dense subgraphs in partition transition of different methods. Because *r*-values are very small when** $p_{out1}/p_{in}\to1$**, the analytical solutions for *Quasi-Surprise* and *Significance* have a large deviation from real ones, the data are obtained by numerical method.** 12](#__RefHeading___Toc484075696)

[**Figure S6. Effect of** $p_{out2}$ **on the critical number of dense subgraphs in partition transition of different methods, in the two-level networks with *p*_out1_/ *p*_in_ =0.2(a), 0.3(b), 0.4(c) and 0.5(d).** 12](#__RefHeading___Toc484075697)

[**Figure S7. The accumulative number of identified communities by different methods in the LFR networks with different network size, different values of k_max_ and C_max_, *k*_m_=10, *C*_min_=20, *µ*=0.1, τ_1_=2, and τ_2_=2. (A) *N*=200 and (B) *N*=500.** 16](#__RefHeading___Toc484075698)

[**Figure S8. Relative values of different quality functions for community partition identified by *Quasi-Surprise*, in the LFR networks with 500 vertices, different mean degrees (*k*_m_), and different community-size heterogeneity. (a) *C*_max_=50 and (b) *C*_max_=100. Other parameters are the same as in Table S1.** 18](#__RefHeading___Toc484075699)

[**Figure S9. Relative values of different quality functions for community partition identified by *Quasi-Surprise*, in the LFR networks with 1000 vertices, different mean degrees (*k*_m_), and different community-size heterogeneity. (a) *C*_max_=50 and (b) *C*_max_=100. Other parameters are the same as in Table S2.** 18](#__RefHeading___Toc484075700)

[**Figure S10. The normalized mutual information (NMI) of community structures identified by different methods, in the LFR networks with 500 vertices, different mean degrees (*k*_m_) and different community-size heterogeneity. Other parameters are the same as in Table S1. (a) *C*_max_=50 and (b) *C*_max_=100 for NMI. (c) *C*_max_=50 and (d) *C*_max_=100 for 1 minus NMI.** 19](#__RefHeading___Toc484075701)

[**Figure S11. The normalized mutual information (NMI) of community structures identified by different methods, in the LFR networks with 1000 vertices, different mean degrees (*k*_m_) and different community-size heterogeneity. Other parameters are the same as in Table S2. (a) *C*_max_=50 and (b) *C*_max_=100 for NMI. (c) *C*_max_=50 and (d) *C*_max_=100 for 1 minus NMI.** 20](#__RefHeading___Toc484075702)

[**Figure S12. Community partitions identified by multi-scale Significance in (A)-(B) two-level networks with *n*_c_=10 and *r*=20, and (C)-(D) hierarchical networks with 256 vertices and two scale community structures. Nc is the identified number of communities in the networks. NMI-1 denotes the NMI between identified and level-1 partitions. NMI-2 denotes the NMI between identified and level-2 partitions.** 22](#__RefHeading___Toc484075703)

[Figure S13. Community partitions identified by multi-scale Modularity in (A)-(B) two-level networks with *n*_c_=10 and *r*=20, and (C)-(D) hierarchical networks with 256 vertices and two scale community structures. Nc is the identified number of communities in the networks. NMI-1 denotes the NMI between identified and level-1 partitions. NMI-2 denotes the NMI between identified and level-2 partitions. 22](#__RefHeading___Toc484075704)

**All Tables**

[**Table S1. For the single-level community-loop networks with different number of pre-defined communities (dense subgraphs) and different *p*_out_-values (*p*_in_ =0.9), “F1|F2|F3|F4” denotes the ratio of the number of identified communities by different methods (*Sq*, *Sp*, *Sg* and *Q*) respectively, to that of pre-defined communities. F*<1 means there exist dense subgraphs merging. “(f1|f2|f3|f4)” denotes the ratio of the values of quality functions of pre-defined partitions for *Sq, Sp, Sg* and *Q* respectively, to that of partitions with 2 dense subgraphs merging. The pre-defined partitions should be preferred when f*>1 , while partitions with dense subgraphs merging should be preferred when f*<1.** 13](#__RefHeading___Toc505456352)

[**Table S2. For the two-level community-loop networks with different number of pre-defined communities (dense subgraphs) and different *p*_out1_-values (*p*_in_=1.0 and *p*_out2_=0.1), “F1|F2|F3|F4” denotes the ratio of the number of identified communities by different methods (*Sq*, *Sp*, *Sg* and *Q*) respectively, to that of pre-defined communities. F*<1 means there exist dense subgraphs merging. “(f1|f2|f3|f4)” denotes the ratio of the values of quality functions of pre-defined partitions for *Sq*, *Sp*, *Sg* and *Q* respectively, to that of partitions with 2 dense subgraphs merging. The pre-defined partitions should be preferred when f*>1 , while partitions with dense subgraphs merging should be preferred when f*<1.** 13](#__RefHeading___Toc505456353)

[**Table S3. For the two-level community-loop networks with different number of pre-defined communities (dense subgraphs) and different *p*_out1_-values (*p*_in_=0.9 and *p*_out2_=0.1), “F1|F2|F3|F4” denotes the ratio of the number of identified communities by different methods (*Sq*, *Sp*, *Sg* and *Q*) respectively, to that of pre-defined communities. F*<1 means there exist dense subgraphs merging. “(f1|f2|f3|f4)” denotes the ratio of the values of quality functions of pre-defined partitions for *Sq*, *Sp*, *Sg* and *Q* respectively, to that of partitions with 2 dense subgraphs merging. The pre-defined partitions should be preferred when f*>1 , while partitions with dense subgraphs merging should be preferred when f*<1.** 14](#__RefHeading___Toc505456354)

[**Table S4. The normalized mutual information (NMI) of community structures identified by different methods, in the LFR networks with different network size (N), different heterogeneity of vertex degrees and community sizes. Other parameters: *k*_m_=10, *C*_min_=20, *µ*=0.1, τ_1_=2, and τ_2_=2. The increase of *k_max_* and *C_max_* will respectively lead the increase of the heterogeneity of degree and community size in the networks.** 16](#__RefHeading___Toc505456355)

[**Table S5. The relative number of communities identified by different methods, compared to the predefined ones, in the LFR networks with different values of *k*_m_ and *C*_max_. Other parameters: *N*=500, *k*_max_=50, *C*_min_=20, *µ*=0.1, τ_1_=2, and τ_1_=2.** 17](#__RefHeading___Toc505456356)

[**Table S6. The relative number of communities identified by different methods, compared to the predefined ones, in the LFR networks with different values of *k*_m_ and *C*_max_. Other parameters: *N*=1000, *k*_max_=50, *C*_min_=20, *µ*=0.1, τ_1_=2, and τ_1_=2.** 17](#__RefHeading___Toc505456357)

[Table S7. The number of communities in various real-world networks, identified by different methods. 23](#__RefHeading___Toc505456358)

## **1. Effect of network parameters on original Surprise, Significance and Modularity**

Here, **Figure S1**, **Figure S2** and **Figure S3** further exhibit the effect of network parameters on other quality functions, original Surprise, Significance and Modularity. The behaviors of Modularity have been widely investigated in many literatures .

**Figure S1**. Relation between *S* (Q) and *x* in distinct networks, normalized by the *S*(Q)-values of the pre-defined partition (*x*=1). (A) original Surprise (*p*_in_=1 and *p*_out_=0.25), (B) Significance (*p*_in_=1 and *p*_out_=0.25), and (C) Modularity (*p*_in_=1 and *p*_out_=0.1).

**Figure S2.** Relation between *S*(Q) and *r* for distinct *x*-values, normalized by the *S*(Q)-values of the pre-defined partition. (A) original Surprise (*p*_in_=1 and *p*_out_=0.25), (B) Significance (*p*_in_=1 and *p*_out_=0.25), and (C) Modularity (*p*_in_=1 and *p*_out_=0.1).

**Figure S3.** Relation between *S*(Q) and $p_{out}\text{/}p_{in}$ for distinct *x*-values (m is the number of edges in the networks). (A) original Surprise, (B) Significance, and (C) Modularity.

## **2. Critical behaviors of Quasi-Surprise, Modularity, Significance and original Surprise on single-level networks**

### **2.1 Critical number of dense subgraphs for Quasi-Surprise**

Here, we derive the critical number $r_{x_{1}\to x_{2}}^{*}$ of dense subgraphs from Partition X_1_ to Partition X_2_ (Partition X denotes the partition with *r/x* groups of *x* dense subgraphs merging). When $\Delta S=S(x_{2})-S(x_{1})>0$, Partition X_2_ will be preferred, compared to Partition X_1_.

$\begin{matrix} \Delta S=\frac{S(x_{2})-S(x_{1})}{m}=q_{x_{1}}\ln\left(\frac{q_{x_{2}}}{\bar{q}_{x_{2}}}\frac{\bar{q}_{x_{1}}}{q_{x_{1}}}\right)+(1-q_{x_{1}})\ln\left(\frac{1-q_{x_{2}}}{1-\bar{q}_{x_{2}}}\frac{1-\bar{q}_{x_{1}}}{1-q_{x_{1}}}\right)+\beta\ln\left(\frac{q_{x_{2}}}{\bar{q}_{x_{2}}}\frac{1-\bar{q}_{x_{2}}}{1-q_{x_{2}}}\right) \\ =q_{x_{1}}\ln\left(\frac{q_{x_{2}}}{q_{x_{1}}}\frac{x_{1}}{x_{2}}\right)+(1-q_{x_{1}})\ln\left(\frac{1-q_{x_{2}}}{1-q_{x_{1}}}\frac{1-\frac{x_{1}}{r}}{1-\frac{x_{2}}{r}}\right)+\beta\ln\left(\frac{q_{x_{2}}}{1-q_{x_{2}}}\frac{1-\frac{x_{2}}{r}}{\frac{x_{2}}{r}}\right) \\ =\frac{p_{in}+\frac{(x_{1}-1)}{x_{1}}2p_{out}}{p_{in}+2p_{out}}\ln\left(\frac{p_{in}+\frac{(x_{2}-1)}{x_{2}}2p_{out}}{p_{in}+\frac{(x_{1}-1)}{x_{1}}2p_{out}}\frac{x_{1}}{x_{2}}\right)+\frac{\frac{2p_{out}}{x_{1}}}{p_{in}+2p_{out}}\ln\left(\frac{\frac{2p_{out}}{x_{2}}}{\frac{2p_{out}}{x_{1}}}\frac{1-\frac{x_{1}}{r}}{1-\frac{x_{2}}{r}}\right) \\ +\left(\frac{x_{2}-1}{x_{2}}-\frac{x_{1}-1}{x_{1}}\right)\frac{2p_{out}}{p_{in}+2p_{out}}\ln\left(\frac{p_{in}+\frac{(x_{2}-1)}{x_{2}}2p_{out}}{\frac{2p_{out}}{x_{2}}}\frac{1-\frac{x_{2}}{r}}{\frac{x_{2}}{r}}\right) \\ \approx q_{x_{1}}\ln\left(\frac{q_{x_{2}}}{q_{x_{1}}}\frac{x_{1}}{x_{2}}\right)+(1-q_{x_{1}})\ln\left(\frac{1-q_{x_{2}}}{1-q_{x_{1}}}\right)+\beta\ln\left(\frac{q_{x_{2}}}{1-q_{x_{2}}}\frac{r}{x_{2}}\right) \\ \approx-D(q_{x_{1}}||q_{x_{2}})+q_{x_{1}}\ln\left(\frac{x_{1}}{x_{2}}\right)+\beta\ln\left(\frac{q_{x_{2}}}{1-q_{x_{2}}}\frac{r}{x_{2}}\right) \end{matrix}$,

where $\beta=\left(\frac{x_{2}-1}{x_{2}}-\frac{x_{1}-1}{x_{1}}\right)\frac{2p_{out}}{p_{in}+2p_{out}}$; for $x_{1}=1$, $\beta=\frac{x_{2}-1}{x_{2}}\frac{2p_{out}}{p_{in}+2p_{out}}$.

By solving $\Delta S=0$ for *r*, one can obtain,

$$\begin{matrix} r_{x_{1}\to x_{2}}^{*}\approx\frac{1-q_{x_{2}}}{q_{x_{2}}}\exp\left(\frac{1}{\beta}D(q_{x_{1}}||q_{x_{2}})\right)\left(\frac{x_{2}}{x_{1}}\right)^{\frac{q_{x_{1}}}{\beta}}\cdot x_{2} \\ \text{=}\frac{\frac{2p_{out}}{x_{2}}}{p_{in}+2p_{out}\frac{(x_{2}-1)}{x_{2}}}\exp\left\{\frac{1}{2p_{out}\left(\frac{x_{2}-1}{x_{2}}-\frac{x_{1}-1}{x_{1}}\right)}\left[\left(p_{in}+2p_{out}\frac{(x_{1}-1)}{x_{1}}\right)\ln\frac{p_{in}+2p_{out}\frac{(x_{1}-1)}{x_{1}}}{p_{in}+2p_{out}\frac{(x_{2}-1)}{x_{2}}}+\frac{2p_{out}}{x_{1}}\ln\frac{\frac{2p_{out}}{x_{1}}}{\frac{2p_{out}}{x_{2}}}\right]\right\} \\ \left(\frac{x_{2}}{x_{1}}\right)^{\frac{p_{in}+2p_{out}\frac{(x_{1}-1)}{x_{1}}}{2p_{out}\left(\frac{x_{2}-1}{x_{2}}-\frac{x_{1}-1}{x_{1}}\right)}} \cdot x_{2} \\ \text{=}\frac{\frac{2p_{out}}{x_{2}}}{p_{in}+2p_{out}\frac{(x_{2}-1)}{x_{2}}}\exp\left\{\frac{p_{in}+2p_{out}\frac{(x_{1}-1)}{x_{1}}}{2p_{out}\left(\frac{x_{2}-1}{x_{2}}-\frac{x_{1}-1}{x_{1}}\right)}\ln\frac{p_{in}+2p_{out}\frac{(x_{1}-1)}{x_{1}}}{p_{in}+2p_{out}\frac{(x_{2}-1)}{x_{2}}}+\frac{1}{x_{1}\left(\frac{x_{2}-1}{x_{2}}-\frac{x_{1}-1}{x_{1}}\right)}\ln\frac{x_{2}}{x_{1}}\right\} \\ \left(\frac{x_{2}}{x_{1}}\right)^{\frac{p_{in}+2p_{out}\frac{(x_{1}-1)}{x_{1}}}{2p_{out}\left(\frac{x_{2}-1}{x_{2}}-\frac{x_{1}-1}{x_{1}}\right)}} \cdot x_{2} \end{matrix}$$

### **2.2 Critical number of dense subgraphs for Modularity**

For comparison, the critical point for Modularity for *r/x* groups of *x* dense subgraphs merging is derived in the networks. For Partiton X,

$\begin{matrix} Q_{x}=\frac{r}{x}\left(\frac{xp_{in}+2(x-1)p_{out}}{r\cdot p_{in}+2r\cdot p_{out}}-\left(\frac{x}{r}\right)^{2}\right) \\ \text{=}\frac{p_{in}+2\frac{x-1}{x}p_{out}}{p_{in}+2p_{out}}-\frac{x}{r} \end{matrix}$.

By solving $Q_{x_{2}}-Q_{x_{1}}=0$ for *r*, one can obtain,

$r_{x_{1}\to x_{2}}^{*}=\frac{p_{in}+2p_{out}}{2p_{out}}\frac{x_{2}-x_{1}}{\left(\frac{x_{2}-1}{x_{2}}-\frac{x_{1}-1}{x_{1}}\right)}$.

For $x_{1}=1$, $r_{x}^{*}=\frac{p_{in}+2p_{out}}{2p_{out}}x$.

### **2.3 Critical number of dense subgraphs for Significance**

For comparison, we show the critical number $r_{2}^{*}$ of dense subgraphs from Partition X=1 to Partition X=2. When $\Delta S=S_{g}(x_{2})-S_{g}(x_{1})>0$, Partition X_2_ will be preferred, compared to Partition X_1_.

The critical number of dense subgraphs for Significance in the partition transition reads,

$r_{2}^{*}=p'\cdot\exp\left(\frac{2H(p_{2})-H(p_{1})}{2p_{2}-p_{1}}\right)$,

Where $H(x)=-x\ln\left(x\right)-(1-x)\ln\left(1-x\right)$, $\Delta H=H(p_{2})-H(p_{1})$, $\Delta p=p_{2}-p_{1}$and $p'=p_{i}+2p_{o}$. See Equations - for the proof of the equation when $p_{out1}=p_{out2}=p_{out}$.

### **2.4 Critical number of dense subgraphs for original Surprise**

In the networks, it is by numerical method that the critical number of dense subgraphs in partition transition is obtained, because the nonlinearity of Surprise leads to the difficulty of analytically deriving the critical number of dense subgraphs.

## **3. Test of “potential well” effect for original Surprise and Significance**

For original Surprise and Significance, in **Figure S4**, we also provided the increment of *S*, $(S-S_{0})/m$, as a function of the fraction of generating 2-community groups, normalized by the number of edges in networks. $S_{0}$ is the *S*-value of the original partition.

In the test, original Surprise shows the “potential well” effect, and Significance does not (see Equation for the proof of Significance).

**Figure S4.** The increase of *S*, $(S-S_{0})/m$, as a function of the fraction of generating 2-community groups in networks with different number of dense subgraphs. $S_{0}$ is the *S*-value of the original partition. *p*_in_=1, *p*_out_=0.25. (a)-(c) for Surprise. (d)-(f) for Significance.

## **4. Critical behaviors of Quasi-Surprise, Modularity, Significance and original Surprise on networks with double-level community structures**

To further analyze the problems in the merging/splitting of communities, we further constructed the two-level networks, which are a generalization of the single-scale networks. Let *r* the number of communities and *n*_c_ the number of vertices in each community at the first level, while $n=r\cdot n_{c}$ is the number of vertices in the network. $p_{in}$ is the probability of linking vertices within the first-level community; $p_{out1}$ is the probability of linking vertices respectively in two first-level and adjacent communities contained in the same second-level community; $p_{out2}(<p_{out1})$ is the probability of linking vertices respectively that belong to two adjacent and first-level communities but are contained in two different second-level communities; the number of edges in the networks is $m=r\cdot n_{c}^{2}p_{in}+r\cdot n_{c}^{2}p_{out1}+r\cdot n_{c}^{2}p_{out2}$. We derived the critical number $r_{2}^{*}$ of communities from Partition X=1 to Partition X=2. See Figure S5 and Figure S6 for the illustration of the critical behaviors.

### **4.1 Critical number of communities for Quasi-Surprise**

In the two-level networks, for the first level,

$\begin{matrix} q_{1}=\frac{r\cdot n_{c}^{2}p_{in}}{r\cdot n_{c}^{2}p_{in}+r\cdot n_{c}^{2}p_{out1}+r\cdot n_{c}^{2}p_{out2}} \\ =\frac{p_{in}}{p_{in}+p_{out1}+p_{out2}} \end{matrix}$ and $\overline{q}_{1}=\frac{r\cdot n_{c}^{2}}{(r\cdot n_{c})^{2}}=\frac{1}{r}$.

So $S_{1}=mD(q_{1}||\bar{q}_{1})$. For the second level,

$\begin{matrix} q_{2}=\frac{r\cdot n_{c}^{2}p_{in}+r\cdot n_{c}^{2}p_{out1}}{r\cdot n_{c}^{2}p_{in}+r\cdot n_{c}^{2}p_{out1}+r\cdot n_{c}^{2}p_{out2}} \\ =\frac{p_{in}+p_{out1}}{p_{in}+p_{out1}+p_{out2}} \end{matrix}$ and $\overline{q}_{2}=\frac{2r\cdot n_{c}^{2}}{(r\cdot n_{c})^{2}}=\frac{2}{r}$.

So $S_{2}=mD(q_{2}||\bar{q}_{2})$. If $S_{2}-S_{1}>0$, the second level is preferred, otherwise the opposite. By solving $S_{2}-S_{1}=0$ for *r*, one can obtain the critical value,

$\begin{matrix} r_{2}^{*}\approx\frac{1-q_{2}}{q_{2}}\cdot\exp\{\frac{1}{\beta}D(q_{1}||q_{2})\}\cdot2^{\frac{q_{1}}{\beta}+1} \\ =\frac{p_{out2}}{p_{in}+p_{out1}}\cdot\exp\left(\frac{p_{in}}{p_{out1}}\ln\frac{p_{in}}{p_{in}+p_{out1}}+\frac{p_{out1}+p_{out2}}{p_{out1}}\ln\frac{p_{out1}+p_{out2}}{p_{out2}}\right)\cdot2^{\frac{p_{in}}{p_{out1}}+1} \\ =\frac{p_{out2}}{p_{in}+p_{out1}}\cdot\left(\frac{p_{in}}{p_{in}+p_{out1}}\right)^{\frac{p_{in}}{p_{out1}}}\left(\frac{p_{out1}+p_{out2}}{p_{out2}}\right)^{\frac{p_{out1}+p_{out2}}{p_{out1}}}\cdot2^{\frac{p_{in}}{p_{out1}}+1} \end{matrix}$,

where $\beta=\frac{p_{out1}}{p_{in}+p_{out1}+p_{out2}}$.

Similarly, we also consider only one group of 2 communities merging in the networks.$\Delta q_{2'}=\frac{p_{out1}}{p_{in}+p_{out1}+p_{out2}}\cdot\frac{2}{r}$ and $\Delta\overline{q}_{2'}=\frac{2}{r^{2}}$. By solving $\Delta S=\frac{\partial S}{\partial q}\Delta q+\frac{\partial S}{\partial\bar{q}}\Delta\bar{q}=0$ for *r*,

$r_{2'}^{*}\approx\frac{p_{out1}+p_{out2}}{p_{in}}\exp\left(\frac{p_{in}}{p_{out1}}\right)$.

When $p_{out1}=p_{out2}$, the networks are the same as the above-mentioned single-scale networks. When $p_{out1}>p_{out2}$, the two-scale structures emerge. With the decrease of $p_{out2}/p_{in}$, the critical $r_{2}^{*}$-values decrease for corresponding parameters. If $p_{out2}=0$, $r_{2}^{*}\approx\frac{p_{out1}}{p_{in}}\cdot\left(\frac{2p_{in}}{p_{in}+p_{out1}}\right)^{\frac{p_{in}}{p_{out1}}+1}$, which means that the effect of $p_{out2}/p_{in}$ on *r* is very limited. The values of $r_{2}$ is mainly determined by $p_{out1}/p_{in}$. Also, Equation has similar behaviors.

### **4.2 Critical number of communities for Modularity**

In the networks, we also analyzed the critical point for Modularity. For the first level, $k_{s}=n_{c}^{2}p_{in}+n_{c}^{2}p_{out1}+n_{c}^{2}p_{out2}$, $k_{s}^{in}=n_{c}^{2}p_{in}$ and

$\begin{matrix} Q_{1}=r\left\{\frac{n_{c}^{2}p_{in}}{r(n_{c}^{2}p_{in}+n_{c}^{2}p_{out1}+n_{c}^{2}p_{out2})}-\left[\frac{n_{c}^{2}p_{in}+n_{c}^{2}p_{out1}+n_{c}^{2}p_{out2}}{r(n_{c}^{2}p_{in}+n_{c}^{2}p_{out1}+n_{c}^{2}p_{out2})}\right]^{2}\right\} \\ =\frac{p_{in}}{p_{in}+p_{out1}+p_{out2}}-\frac{1}{r} \end{matrix}$.

For the second level, $k_{s2}=2n_{c}^{2}p_{in}+2n_{c}^{2}p_{out1}+2n_{c}^{2}p_{out2}=2k_{s}$, $k_{s2}^{in}=2n_{c}^{2}p_{in}+2n_{c}^{2}p_{out1}$ and

$\begin{matrix} Q_{2}=\frac{r}{2}\left\{\frac{2n_{c}^{2}p_{in}+2n_{c}^{2}p_{out1}}{r(n_{c}^{2}p_{in}+n_{c}^{2}p_{out1}+n_{c}^{2}p_{out2})}-\left[\frac{2n_{c}^{2}p_{in}+2n_{c}^{2}p_{out1}+2n_{c}^{2}p_{out2}}{r(n_{c}^{2}p_{in}+n_{c}^{2}p_{out1}+n_{c}^{2}p_{out2})}\right]^{2}\right\} \\ =\frac{p_{in}+p_{out1}}{p_{in}+p_{out1}+p_{out2}}-\frac{2}{r} \end{matrix}$.

By solving $Q_{2}-Q_{1}=0$ for *r*,

$r_{2}^{*}=\frac{p_{in}+p_{out1}+p_{out2}}{p_{out1}}$,

It is clear that Modularity finds the second level when $r>r_{2}^{*}$, otherwise the first level is found. Because of the linear property of Modularity, it has the same critical $r_{2}^{*}$-value for *r/*2 groups and single group of 2 communities merging. Consider there are *t* groups of 2 communities merging,

$\begin{matrix} Q_{2}(t)=(r-2t)\left\{\frac{n_{c}^{2}p_{in}}{r(n_{c}^{2}p_{in}+n_{c}^{2}p_{out1}+n_{c}^{2}p_{out2})}-\left[\frac{n_{c}^{2}p_{in}+n_{c}^{2}p_{out1}+n_{c}^{2}p_{out2}}{r(n_{c}^{2}p_{in}+n_{c}^{2}p_{out1}+n_{c}^{2}p_{out2})}\right]^{2}\right\} \\ +t\left\{\frac{2n_{c}^{2}p_{in}+2n_{c}^{2}p_{out1}}{r(n_{c}^{2}p_{in}+n_{c}^{2}p_{out1}+n_{c}^{2}p_{out2})}-\left[\frac{2n_{c}^{2}p_{in}+n_{c}^{2}p_{out1}+n_{c}^{2}p_{out2}}{r(n_{c}^{2}p_{in}+n_{c}^{2}p_{out1}+n_{c}^{2}p_{out2})}\right]^{2}\right\} \\ =(r-2t)(\frac{1}{r}\frac{p_{in}}{p_{in}+p_{out1}+p_{out2}}-\frac{1}{r^{2}})+t(\frac{2}{r}\frac{p_{in}+p_{out1}}{p_{in}+p_{out1}+p_{out2}}-\frac{4}{r^{2}}) \\ =r(\frac{1}{r}\frac{p_{in}}{p_{in}+p_{out1}+p_{out2}}-\frac{1}{r^{2}})+t(\frac{2}{r}\frac{p_{in}+p_{out1}}{p_{in}+p_{out1}+p_{out2}}-\frac{4}{r^{2}}-2(\frac{1}{r}\frac{p_{in}}{p_{in}+p_{out1}+p_{out2}}-\frac{1}{r^{2}})) \\ =(\frac{p_{in}}{p_{in}+p_{out1}+p_{out2}}-\frac{1}{r})+t(\frac{2}{r}\frac{p_{out1}}{p_{in}+p_{out1}+p_{out2}}-\frac{2}{r^{2}}) \\ =(\frac{p_{in}}{p_{in}+p_{out1}+p_{out2}}-\frac{1}{r})+\frac{t}{r}(\frac{p_{out1}}{p_{in}+p_{out1}+p_{out2}}-\frac{1}{r}) \end{matrix}$.

Clearly, $Q_{2}(t)$ is a monotonically increasing function. When $(\frac{p_{out1}}{p_{in}+p_{out1}+p_{out2}}-\frac{1}{r})>0$, communities merging leads that $Q_{2}(t)$ increases with *t.* Because of the accumulative property of Modularity, it does not show the potential-well phenomenon.

### **4.3 Critical number of communities for Significance**

For the first-level partition, there are *r* communities, each of which has $n_{c}$ nodes,

$p_{1}=\frac{n_{c}^{2}p_{in}}{n_{c}^{2}}=p_{in}$ and $p=\frac{r\cdot n_{c}^{2}p_{in}+r\cdot n_{c}^{2}p_{out1}+r\cdot n_{c}^{2}p_{out2}}{\left(r\cdot n_{c}\right)^{2}}=\frac{p_{in}+p_{out1}+p_{out2}}{r}$,

$\begin{matrix} Sg1=r\frac{n_{c}(n_{c}-1)}{2}D(p_{1}||p) \\ =r\frac{n_{c}(n_{c}-1)}{2}\left(p_{1}\ln\frac{p_{1}}{p}+(1-p_{1})\ln\frac{1-p_{1}}{1-p}\right) \end{matrix}$,

For the second-level partition, there are r/2 communities, each of which has $2n_{c}$ nodes,

$p_{2}=\frac{2n_{c}^{2}p_{in}+2n_{c}^{2}p_{out1}}{\left(2n_{c}\right)^{2}}=\frac{2p_{in}+2p_{out1}}{4}=\frac{p_{in}+p_{out1}}{2}$ and $p=\frac{p_{in}+p_{out1}+p_{out2}}{r}$,

$\begin{matrix} Sg2=\frac{r}{2}\frac{2n_{c}(2n_{c}-1)}{2}D(p_{2}||p) \\ =\frac{r}{2}\frac{2n_{c}(2n_{c}-1)}{2}\left(p_{2}\ln\frac{p_{2}}{p}+(1-p_{2})\ln\frac{1-p_{2}}{1-p}\right) \\ \approx r\cdot n_{c}^{2}\left(p_{2}\ln\frac{p_{2}}{p}+(1-p_{2})\ln\frac{1-p_{2}}{1-p}\right) \end{matrix}$,

When $Sg2-Sg1>0$, the second-level partition should be identified. In this case,

$\left(\left(\begin{matrix} 2n_{c} \\ 2 \end{matrix}\right)D(p_{2}||p)-2\left(\begin{matrix} n_{c} \\ 2 \end{matrix}\right)D(p_{1}||p)\right)>0$.

Moreover, consider there are *t* groups of 2 communities merging,

$\begin{matrix} Sg2(t)=(r-2t)\left(\begin{matrix} n_{c} \\ 2 \end{matrix}\right)D(p_{1}||p)+t\left(\begin{matrix} 2n_{c} \\ 2 \end{matrix}\right)D(p_{2}||p) \\ =\left(\left(\begin{matrix} 2n_{c} \\ 2 \end{matrix}\right)D(p_{2}||p)-2\left(\begin{matrix} n_{c} \\ 2 \end{matrix}\right)D(p_{1}||p)\right)t+r\left(\begin{matrix} n_{c} \\ 2 \end{matrix}\right)D(p_{1}||p) \\ =\left(\left(\begin{matrix} 2n_{c} \\ 2 \end{matrix}\right)D(p_{2}||p)-2\left(\begin{matrix} n_{c} \\ 2 \end{matrix}\right)D(p_{1}||p)\right)t+r\left(\begin{matrix} n_{c} \\ 2 \end{matrix}\right)D(p_{1}||p) \end{matrix}$.

Clearly, $Sg2(t)$ is a monotonically increasing function. When $\left(\left(\begin{matrix} 2n_{c} \\ 2 \end{matrix}\right)D(p_{2}||p)-2\left(\begin{matrix} n_{c} \\ 2 \end{matrix}\right)D(p_{1}||p)\right)>0$, which is the same as the above inequality, communities merging leads that $Sg2(t)$ increases with $t$. Because of the accumulative property of Significance, it does not show the potential-well phenomenon.

By solving equation for *r*, the critical number of communities for Significance,

$r_{2}^{*}=p'\cdot\exp\left(\frac{2H(p_{2})-H(p_{1})}{2p_{2}-p_{1}}\right)$,

Where $H(y)=-y\ln\left(y\right)-(1-y)\ln\left(1-y\right)$, $p_{1}=p_{in}$, $p_{2}=\left(p_{in}+p_{out1}\right)/2$, $p'=p_{in}+p_{out1}+p_{out2}$.

**Proof.** Suppose that $n_{c}-1\approx n_{c}$, $2n_{c}-1\approx2n_{c}$, $1\text{-}p\approx1$ for large *r*–values, and define $p=p'/r$. By equation

$$0\text{=}\frac{2n_{c}(2n_{c}-1)}{2}D(p_{2}||p)-2\frac{n_{c}(n_{c}-1)}{2}D(p_{1}||p)$$

$$0\text{=}2\left(p_{2}\ln\left(\frac{p_{2}}{p'}r\right)+(1-p_{2})\ln\left(1-p_{2}\right)\right)-\left(p_{1}\ln\left(\frac{p_{1}}{p'}r\right)+(1-p_{1})\ln\left(1-p_{1}\right)\right)$$

$$\left(2p_{2}-p_{1}\right)\ln\left(r\right)=p_{1}\ln\left(p_{1}\right)+(1-p_{1})\ln\left(1-p_{1}\right)-2\left(p_{2}\ln p_{2}+(1-p_{2})\ln\left(1-p_{2}\right)\right)+(2p_{2}-p_{1})\ln p'$$

$\begin{matrix} r=\exp\left(\frac{1}{2p_{2}-p_{1}}\left(p_{1}\ln\left(\frac{p_{1}}{p'}\right)+(1-p_{1})\ln\left(1-p_{1}\right)-2\left(p_{2}\ln\left(\frac{p_{2}}{p'}\right)+(1-p_{2})\ln\left(1-p_{2}\right)\right)\right)+\ln p'\right) \\ =p'\cdot\exp\left(\frac{2H(P_{2})-H(P_{1})}{2p_{2}-p_{1}}\right) \end{matrix}$,

For illustration, **Figure S5** displays the relation between the critical number $r_{2}^{*}$ of communities and network parameters. We can see that $r_{2}^{*}$ decreases with the increase of $p_{out1}$. This is reasonable, because the increase of the number of links between communities will make the communities merging more easily. For large $p_{out1}$-values, the $r_{2}^{*}$-values are very small, which are close to that of Modularity. However, for small $p_{out1}$-values, the $r_{2}^{*}$-values dramatically increase with the decrease of $p_{out1}$, which is far greater than that of Modularity. As a result, Significance generally tends to split the communities in the networks, especially with small inter-community link density, and find more communities than other methods, such as Modularity. This is also confirmed by the experimental results.

### **4.4 Critical number of communities for original Surprise**

In the two-level networks, because of the nonlinearity of Surprise, it is by numerical method that the critical number of communities from the first-level Partition to the second-level Partition is obtained.

**Figure S5.** In the two-level networks, effect of $p_{out1}/p_{in}$ on the critical number of communities in partition transition of different methods. Note that the *r*-values are very small when $p_{out1}/p_{in}\to1$, the analytical solutions for Quasi-Surprise and Significance have a large deviation from real ones, so the data are obtained by numerical method.

**Figure S6.** Effect of $p_{out2}$ on the critical number of communities in partition transition of different methods, in the two-level networks with *p*_out1_/ *p*_in_ =0.2(a), 0.3(b), 0.4(c) and 0.5(d).

## **5. Experimental results based on direct optimization**

### **5.1 Test in community-loop networks with single- and two-level community structures**

**Table S1.** “**F1|F2|F3|F4**” (the numbers separated by vertical lines) denotes the ratio of the number of identified communities by *Sq*, *Sp*, *Sg* and *Q* respectively, to that of pre-defined communities (i.e. dense subgraphs), in the single-level networks with different number of pre-defined communities and different *p*_out_-values (*p*_in_=0.9). “(**f1|f2|f3|f4)**” (the numbers in the parentheses, separated by vertical lines) denotes the ratio of the values of quality functions of pre-defined partitions for *Sq*, *Sp*, *Sg* and *Q* respectively, to that of Partition X=2. If F1, F2, F3 or F4 is less than a value of 1, then there are the appearance of communities merging, that is, there are at least 2 or more communities being merged into a large community by corresponding methods (Sq, Sp, Sg or *Q*). If f1, f2, f3 or f4 is less than a value of 1, then Partition X=2 should be preferred by Sq, Sp, Sg or *Q*, but the identified partitions are not inevitably to be Partition X=2 or the partition with communities merging.

| ***p*_out_** | | **Number of pre-defined communities in single-level networks**  **4 8 16 32** | | | |
| --- | --- | --- | --- | --- | --- |
|  | **0.1** | **1.00**|**1.00**|**1.00**|**1.00** (1.79|2.28|3.91|1.37) | **1.00**|**1.00**|**1.00**|**1.00** (1.26|1.51|2.01|1.04) | **1.00**|**1.00**|**1.00**|0.55 (1.11|1.30|1.55|0.95) | **1.00**|**1.00**|**1.00**|0.43 (1.05|1.19|1.35|0.92) |
|  | **0.2** | **1.00**|**1.00**|**1.00**|**1.00** (1.61|2.01|3.50|1.26) | **1.00**|**1.00**|**1.00**|0.58 (1.06|1.30|1.70|0.93) | **0.96**|**1.00**|**1.00**|0.41 (**0.95**|1.12|1.33|0.86) | 0.57|**1.00**|**1.00**|0.32 (0.90|1.03|1.17|0.83) |
|  | **0.3** | **1.00**|**1.00**|**1.00**|0.85 (1.39|1.81|3.03|1.14) | 0.65|**1.00**|**1.00**|0.55 (0.90|1.11|1.44|0.84) | 0.56|**1.00**|**1.00**|0.39 (0.82|**0.95**|1.14|0.77) | 0.44|**1.00**|**1.00**|0.27 (0.79|**0.90**|1.01|0.75) |
|  | **0.4** | **1.00**|**1.00**|**1.00**|0.60 (1.10|1.51|2.42|**1.00**) | 0.55|**1.00**|**1.00**|0.48 (0.76|**0.95**|1.21|0.75) | 0.44|0.83|0.58|0.38 (0.71|0.83|0.98|0.70) | 0.44|0.57|0.58|0.26 (0.69|0.79|0.89|0.68) |
|  | **0.5** | 0.55|**1.00**|**1.00**|0.55 (0.90|1.24|1.98|0.89) | 0.53|0.58|0.99|0.45 (0.64|0.80|1.00|0.68) | 0.44|0.54|0.55|0.33 (0.62|0.72|0.85|0.64) | 0.44|0.46|0.56|0.25 (0.61|0.69|0.78|0.63) |
|  | **0.6** | 0.50|**1.00**|**1.00**|0.50 (0.66|**0.97**|1.49|0.76) | 0.50|0.58|0.58|0.43 (0.54|0.68|0.85|0.62) | 0.43|0.55|0.55|0.31 (0.54|0.63|0.74|0.59) | 0.43|0.45|0.54|0.22 (0.55|0.61|0.69|0.58) |
|  | **0.7** | 0.50|0.50|0.95|0.50 (0.48|0.75|1.11|0.64) | 0.45|0.53|0.53|0.38 (0.46|0.57|0.71|0.56) | 0.46|0.46|0.58|0.33 (0.48|0.55|0.64|0.55) | 0.42|0.45|0.44|0.22 (0.49|0.54|0.61|0.54) |
|  | **0.8** | 0.50|0.50|0.55|0.50 (0.32|0.53|0.76|0.51) | 0.50|0.60|0.53|0.45 (0.39|0.48|0.58|0.50) | 0.44|0.45|0.55|0.34 (0.42|0.48|0.55|0.50) | 0.34|0.44|0.44|0.22 (0.44|0.48|0.54|0.50) |

**Table S2.** For the two-level community-loop networks with different number of pre-defined communities (dense subgraphs) and different *p*_out1_-values (*p*_in_=1.0 and *p*_out2_=0.1), “**F1|F2|F3|F4**” (the numbers separated by vertical lines) denotes the ratio of the number of identified communities by *Sq*, *Sp*, *Sg* and *Q* respectively, to that of pre-defined communities (i.e. dense subgraphs). “(**f1|f2|f3|f4)**” (the numbers in the parentheses, separated by vertical lines) denotes the ratio of the values of quality functions of pre-defined partitions for *Sq*, *Sp*, *Sg* and *Q* respectively, to that of Partition X=2. If F1, F2, F3 or F4 is less than a value of 1, then there are the appearance of communities merging, that is, there are at least 2 or more communities being merged into a large community by corresponding methods. If f1, f2, f3 or f4 is less than a value of 1, then Partition X=2 should be preferred, but the identified partitions are not inevitably to be Partition X=2 or the partition with communities merging.

| ***p*_out1_** | **Number of pre-defined communities in two-level networks**  **4 8 16 32** | | | |
| --- | --- | --- | --- | --- |
| **0.1** | **1.00**|**1.00**|**1.00**|**1.00** (1.82|2.53|4.53|1.38) | **1.00**|**1.00**|**1.00**|**1.00** (1.27|1.66|2.24|1.05) | **1.00**|**1.00**|**1.00**|0.54 (1.13|1.40|1.69|0.96) | **1.00**|**1.00**|**1.00**|0.44 (1.07|1.28|1.45|0.93) |
| **0.2** | **1.00**|**1.00**|**1.00**|**1.00** (1.33|1.94|3.54|1.20) | **1.00**|**1.00**|**1.00**|0.50 (1.02|1.35|1.86|0.94) | **1.00**|**1.00**|**1.00**|0.50 (**0.94**|1.17|1.44|0.87) | 0.50|**1.00**|**1.00**|0.49 (0.90|1.09|1.26|0.84) |
| **0.3** | **1.00**|**1.00**|**1.00**|**1.00** (1.03|1.55|2.82|1.05) | 0.50|**1.00**|**1.00**|0.50 (0.84|1.12|1.56|0.84) | 0.50|**1.00**|**1.00**|0.49 (0.80|**1.00**|1.24|0.79) | 0.50|**1.00**|**1.00**|0.47 (0.78|**0.94**|1.10|0.77) |
| **0.4** | 0.50|**1.00**|**1.00**|0.50 (0.79|1.23|2.23|0.92) | 0.50|**1.00**|**1.00**|0.50 (0.70|**0.94|**1.32|0.76) | 0.50|**1.00**|**1.00**|0.50 (0.69|**0.86**|1.08|0.72) | 0.50|**1.00**|0.50|0.46 (0.68|**0.82**|0.97|0.71) |
| **0.5** | 0.50|**1.00**|**1.00**|0.50 (0.61|**0.98**|1.77|0.81) | 0.50|**1.00**|**1.00**|0.50 (0.59|**0.80**|1.12|0.70) | 0.50|**1.00**|0.50|0.50 (0.60|**0.75**|0.94|0.66) | 0.50|**1.00**|0.50|0.46 (0.60|**0.72**|0.86|0.65) |
| **0.6** | 0.50|**1.00**|**1.00**|0.50 (0.47|**0.78**|1.40|0.71) | 0.50|0.50|0.50|0.50 (0.50|0.68|0.95|0.64) | 0.50|0.50|0.50|0.50 (0.52|0.65|0.82|0.62) | 0.50|0.50|0.50|0.50 (0.54|0.64|0.76|0.61) |
| **0.7** | 0.50|0.50|**1.00**|0.50 (0.38|0.64|1.11|0.63) | 0.50|0.50|0.50|0.50 (0.43|0.58|0.81|0.59) | 0.50|0.50|0.50|0.50 (0.46|0.57|0.72|0.57) | 0.50|0.50|0.50|0.50 (0.48|0.57|0.68|0.57) |
| **0.8** | 0.50|0.50|0.50|0.50 (0.30|0.52|0.87|0.56) | 0.50|0.50|0.50|0.50 (0.37|0.50|0.69|0.54) | 0.50|0.50|0.50|0.50 (0.41|0.50|0.63|0.53) | 0.50|0.50|0.50|0.50 (0.44|0.51|0.61|0.53) |
| **0.9** | 0.50|0.50|0.50|0.50 (0.24|0.41|0.67|0.50) | 0.50|0.50|0.50|0.50 (0.32|0.43|0.58|0.50) | 0.50|0.50|0.50|0.50 (0.37|0.44|0.56|0.50) | 0.50|0.50|0.50|0.50 (0.40|0.45|0.54|0.50) |

**Table S3.** For the two-level community-loop networks with different number of pre-defined communities (dense subgraphs) and different *p*_out1_-values (*p*_in_=0.9 and *p*_out2_=0.1), “**F1|F2|F3|F4**” (the numbers separated by vertical lines) denotes the ratio of the number of identified communities by *Sq*, *Sp*, *Sg* and *Q* respectively, to that of pre-defined communities (i.e. dense subgraphs). “(**f1|f2|f3|f4)**” (the numbers in the parentheses, separated by vertical lines) denotes the ratio of the values of quality functions of pre-defined partitions for *Sq*, *Sp*, *Sg* and *Q* respectively, to that of Partition X=2. If F1, F2, F3 or F4 is less than a value of 1, then there are the appearance of communities merging, that is, there are at least 2 or more communities being merged into a large community by corresponding methods (Sq, Sp, Sg or *Q*). If f1, f2, f3 or f4 is less than a value of 1, then Partition X=2 should be preferred by Sq, Sp, Sg or *Q*, but the identified partitions are not inevitably to be Partition X=2 or the partition with communities merging.

| ***p*_out1_** | | **Number of pre-defined communities in two-level networks**  **4 8 16 32** | | | |
| --- | --- | --- | --- | --- | --- |
|  | **0.1** | **1.00**|**1.00**|**1.00**|**1.00** (1.79|2.27|3.91|1.37) | **1.00**|**1.00**|**1.00**|**1.00** (1.26|1.52|2.01|1.04) | **1.00**|**1.00**|**1.00**|0.55 (1.11|1.30|1.55|0.95) | **1.00**|**1.00**|**1.00**|0.46 (1.05|1.19|1.35|0.92) |
|  | **0.2** | **1.00**|**1.00**|**1.00**|**1.00** (1.31|1.72|3.01|1.18) | **1.00**|**1.00**|**1.00**|0.50 (**0.99**|1.21|1.64|0.92) | **1.00**|**1.00**|**1.00**|0.50 (**0.91**|1.07|1.30|0.85) | 0.50|**1.00**|**1.00**|0.49 (0.88|**1.00**|1.15|0.82) |
|  | **0.3** | **1.00**|**1.00**|**1.00**|0.85 (**0.95**|1.29|2.27|**1.00**) | 0.50|**1.00**|**1.00**|0.50 (0.79|**0.98**|1.34|0.82) | 0.50|**1.00**|**1.00**|0.50 (0.76|**0.89**|1.10|0.77) | 0.50|**1.00**|0.59|0.47 (0.75|**0.85**|0.99|0.75) |
|  | **0.4** | 0.50|**1.00**|**1.00**|0.50 (0.71|**0.99**|1.75|0.87) | 0.50|**1.00**|**1.00**|0.50 (0.65|**0.81**|1.11|0.73) | 0.50|0.50|0.50|0.50 (0.65|0.76|0.95|0.70) | 0.50|0.50|0.50|0.48 (0.65|0.74|0.87|0.68) |
|  | **0.5** | 0.50|0.60|**1.00**|0.50 (0.54|0.77|1.35|0.75) | 0.50|0.50|0.50|0.50 (0.54|0.68|0.94|0.66) | 0.50|0.50|0.50|0.50 (0.56|0.65|0.82|0.64) | 0.50|0.50|0.50|0.49 (0.57|0.64|0.76|0.63) |
|  | **0.6** | 0.50|0.50|**1.00**|0.50 (0.42|0.61|1.05|0.66) | 0.50|0.50|0.50|0.50 (0.46|0.57|0.79|0.60) | 0.50|0.50|0.50|0.50 (0.48|0.57|0.71|0.59) | 0.50|0.50|0.50|0.50 (0.50|0.57|0.67|0.58) |
|  | **0.7** | 0.50|0.50|0.50|0.50 (0.32|0.48|0.82|0.58) | 0.50|0.50|0.50|0.50 (0.39|0.48|0.66|0.55) | 0.50|0.50|0.50|0.50 (0.43|0.49|0.62|0.54) | 0.50|0.50|0.50|0.50 (0.45|0.50|0.60|0.54) |
|  | **0.8** | 0.50|0.50|0.50|0.50 (0.25|0.38|0.62|0.51) | 0.50|0.50|0.50|0.50 (0.33|0.41|0.56|0.50) | 0.50|0.50|0.50|0.50 (0.38|0.43|0.54|0.50) | 0.50|0.50|0.50|0.50 (0.40|0.44|0.53|0.50) |

Here, we further provide the results on the single-level and two-level community-loop networks, obtained by directly optimizing the corresponding quality functions.

Table S1 shows the further results on the single-level community-loop networks with different number of pre-defined communities and different *p*_out_-values (*p*_in_ =0.9).

**Table S2** and **Table S3** show the results on the two-level community-loop networks with different number of pre-defined communities and different *p*_out1_-values (*p*_in_=1.0 and *p*_out2_=0.1 as well as *p*_in_=0.9 and *p*_out2_=0.1).

### **5.2 Test in LFR networks: Effect of heterogeneity of degree and community size**

Here, we further provide the results on the LFR networks with different parameters in order to display the effect of heterogeneity of degree and community size.

**(1) *Heterogeneity of degree and community size caused by the increase of maximal degree and maximal community size***

By fixing the mean degree and increasing the maximal degree in the networks, the heterogeneity of degree increases gradually, and by fixing the minimal community size and increasing the maximal community size, the heterogeneity of community size increases gradually.

Figure S7 shows the accumulative number of identified communities by different methods, in the LFR networks with different values of k_max_ and C_max_, *N*=200 and 500, *k*_m_=10, *C*_min_=20, *µ*=0.1, τ_1_=2, and τ_2_=2.

Table S4 shows the normalized mutual information (NMI) of community structures identified by different methods, in the LFR networks with different network size, different heterogeneity of vertex degrees and community sizes.

**(2) *Heterogeneity of degree and community size caused by mean degrees and maximal community size***

By fixing the maximal degree and decreasing the mean degree in the networks, the heterogeneity of degree increases gradually, and by fixing the minimal community size and increasing the maximal community size, the heterogeneity of community size increases gradually.

Table S5 and Table S6 show the relative number of communities identified by different methods, compared to the predefined partition, in the LFR networks with different mean degrees (*k*_m_), and different community-size heterogeneity.

Figure S8 and **Figure S9** show the relative values of different quality functions for identified community partitions, compared to the predefined partition, in the LFR networks with different mean degrees (*k*_m_), and different community-size heterogeneity.

**Figure S10** and **Figure S11** show the normalized mutual information (NMI) of identified community structures, in the LFR networks with different mean degrees (*k*_m_) and different community-size heterogeneity.

Firstly, we study the effect of heterogeneity of degree and community size, by increasing k_max_ and C_max_. The inhomogeneity of link density in communities will emerge gradually due to the random fluctuations (Table S2**, Figure S7** and Table S4), and thus communities in the networks tend to split. See text for discussion.

Then, by decreasing the mean degree of the networks, the communities in the networks become more and more sparse, and thus the inhomogeneity of link density in communities will emerge gradually due to the random fluctuations of links. When the inhomogeneity of this type is large enough in a community, the community may be split by some community detection methods. Table S5 and Table S6 shows that the partitions by Quasi-Surprise, Surprise and Significance contain more groups of vertices than the pre-defined ones, because they (as well as LP ) tend to split the communities, especially when comparing with other methods (such as Modularity , Infomap , Walktrap and OSLOM ). With the increase of the mean degree, the tendency to split is weakened gradually.

Figure S8 and **Figure S9** confirm that the Quasi-Surprise, Surprise and Significance tend to find non pre-defined community partitions, because the quality functions for identified community partitions (by Quasi-Surprise) are clearly larger than their corresponding values for the pre-defined partitions respectively. With the increase of the mean degree, the difference between identified and pre-defined community partition gradually decreases.

***Figure S10*** (a)-(b) and Figure S11 (a)-(b) exhibit the normalized mutual information of community structures identified by different methods, though we have no intention to compare the performance of various methods, because this is closely related to the networks under study. The results show that the Quasi-Surprise, *Surprise* and *Significance* have relatively lower NMI than many other methods, due to the split of communities. Figure S10 (c)-(d) and Figure S11 (c)-(d) more clearly display the difference between distinct methods. With the increase of the mean degree, NMI gradually increases, and the difference between distinct methods gradually decreases, because of the weakening of the split of communities.

Further, we can find the effect of the network size on the above results. For Quasi-Surprise, Surprise and Significance, with the increase of network size, (1) the tendency to split is also weakened gradually (See Table S5 and Table S6); (2) the difference between identified and pre-defined community partition gradually decreases (See Figure S8, Figure S9**,** Figure S10 and Figure S11); and (3) NMI gradually increases (See Figure S10 and Figure S11).


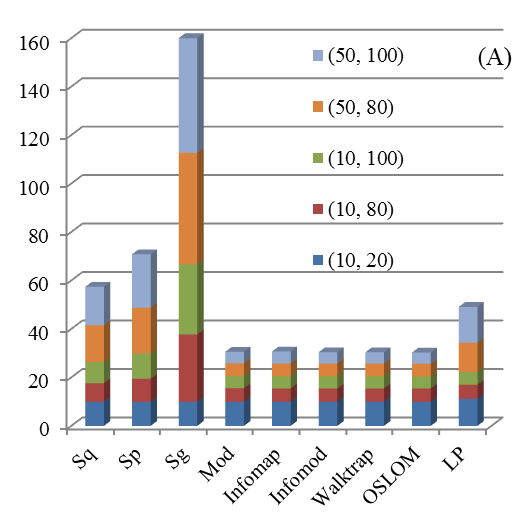

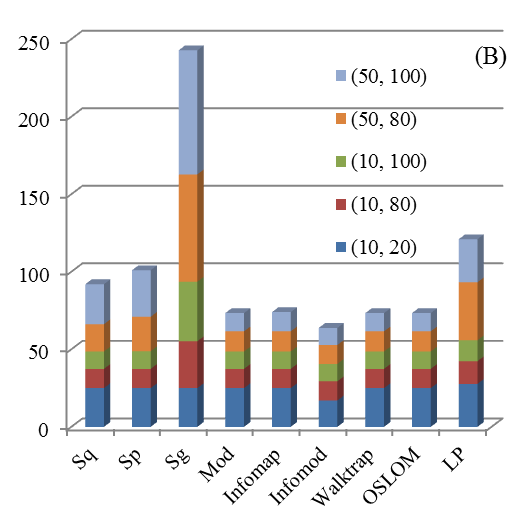


**Figure S7.** The accumulative number of identified communities by different methods in the LFR networks with different network size, different values of k_max_ and C_max_, *k*_m_=10, *C*_min_=20, *µ*=0.1, τ_1_=2, and τ_2_=2. (A) *N*=200 and (B) *N*=500.

**Table S4. The normalized mutual information (NMI) of community structures identified by different methods, in the LFR networks with different network size (N), different heterogeneity of vertex degrees and community sizes. Other parameters: *k*_m_=10, *C*_min_=20, *µ*=0.1, τ_1_=2, and τ_2_=2. The increase of *k_max_* and *C_max_* will respectively lead the increase of the heterogeneity of degree and community size in the networks.**

| ***N*** | ***k_max_*** | ***C_max_*** | ***Sq*** | ***Sp*** | ***Sg*** | ***Mod*** | ***Infomap*** | ***Infomod*** | ***Walktrap*** | ***OSLOM*** | ***LP*** |
| --- | --- | --- | --- | --- | --- | --- | --- | --- | --- | --- | --- |
| 200 | 10 | 10 | 1.00 | 1.00 | 1.00 | 1.00 | 1.00 | 1.00 | 1.00 | 1.00 | 0.99 |
|  |  | 80 | 0.95 | 0.89 | 0.71 | 0.98 | 1.00 | 1.00 | 1.00 | 1.00 | 0.99 |
|  |  | 100 | 0.89 | 0.85 | 0.68 | 1.00 | 1.00 | 1.00 | 1.00 | 0.98 | 0.99 |
|  | 50 | 80 | 0.86 | 0.80 | 0.64 | 1.00 | 1.00 | 0.99 | 1.00 | 1.00 | 0.96 |
|  |  | 100 | 0.82 | 0.73 | 0.59 | 0.99 | 0.99 | 0.96 | 1.00 | 1.00 | 0.92 |
| 500 | 10 | 10 | 1.00 | 1.00 | 1.00 | 1.00 | 1.00 | 0.93 | 1.00 | 1.00 | 0.99 |
|  |  | 80 | 1.00 | 1.00 | 0.89 | 1.00 | 1.00 | 1.00 | 1.00 | 1.00 | 0.99 |
|  |  | 100 | 1.00 | 0.99 | 0.83 | 1.00 | 1.00 | 1.00 | 1.00 | 1.00 | 0.99 |
|  | 50 | 80 | 0.98 | 0.96 | 0.83 | 1.00 | 1.00 | 0.99 | 1.00 | 1.00 | 0.95 |
|  |  | 100 | 0.92 | 0.91 | 0.78 | 1.00 | 1.00 | 0.99 | 1.00 | 1.00 | 0.97 |

**Table S5.** The relative number of communities identified by different methods, compared to the predefined ones, in the LFR networks with different values of *k*_m_ and *C*_max_. Other parameters: *N*=500, *k*_max_=50, *C*_min_=20, *µ*=0.1, τ_1_=2, and τ_1_=2.

| **km** | **Cmax** | **Quasi-Surprise** | **Surprise** | **Significance** | **Modularity** | **Infomap** | **Walktrap** | **OSLOM** | **LP** |
| --- | --- | --- | --- | --- | --- | --- | --- | --- | --- |
| 8 | 50 | 1.24 | 1.49 | 4.26 | 1.00 | 1.01 | 1.00 | 1.00 | 5.72 |
| 12 | 50 | 1.04 | 1.08 | 3.01 | 1.00 | 1.00 | 1.00 | 1.00 | 2.85 |
| 16 | 50 | 1.00 | 1.02 | 2.01 | 1.00 | 1.00 | 1.00 | 1.00 | 1.93 |
| 20 | 50 | 1.00 | 1.00 | 1.42 | 1.00 | 1.00 | 1.00 | 1.00 | 1.14 |
| 8 | 100 | 2.71 | 3.31 | 7.72 | 1.00 | 1.10 | 1.00 | 1.00 | 3.48 |
| 12 | 100 | 1.47 | 1.79 | 5.61 | 1.00 | 1.00 | 1.00 | 1.00 | 1.67 |
| 16 | 100 | 1.09 | 1.22 | 3.32 | 1.00 | 1.00 | 1.00 | 1.00 | 1.26 |
| 20 | 100 | 1.00 | 1.03 | 2.28 | 1.00 | 1.00 | 1.00 | 1.00 | 1.01 |

**Table S6.** The relative number of communities identified by different methods, compared to the predefined ones, in the LFR networks with different values of *k*_m_ and *C*_max_. Other parameters: *N*=1000, *k*_max_=50, *C*_min_=20, *µ*=0.1, τ_1_=2, and τ_1_=2.

| **km** | **Cmax** | **Quasi-Surprise** | **Surprise** | **Significance** | **Modularity** | **Infomap** | **Walktrap** | **OSLOM** | **LP** |
| --- | --- | --- | --- | --- | --- | --- | --- | --- | --- |
| 8 | 50 | 1.13 | 1.20 | 3.10 | 0.99 | 1.00 | 1.00 | 1.00 | 5.54 |
| 12 | 50 | 1.01 | 1.02 | 1.64 | 1.00 | 1.00 | 1.00 | 1.00 | 2.60 |
| 16 | 50 | 1.00 | 1.00 | 1.28 | 1.00 | 1.00 | 1.00 | 1.00 | 1.74 |
| 20 | 50 | 1.00 | 1.00 | 1.10 | 1.00 | 1.00 | 1.00 | 1.00 | 1.10 |
| 8 | 100 | 1.82 | 2.00 | 4.46 | 1.00 | 1.02 | 1.00 | 1.00 | 4.21 |
| 12 | 100 | 1.15 | 1.33 | 2.96 | 1.00 | 1.00 | 1.00 | 1.00 | 1.74 |
| 16 | 100 | 1.02 | 1.05 | 1.59 | 1.00 | 1.00 | 1.00 | 1.00 | 1.29 |
| 20 | 100 | 1.01 | 1.04 | 1.07 | 1.00 | 1.00 | 1.00 | 1.00 | 1.03 |

**Figure S8.** Relative values of different quality functions for community partition identified by *Quasi-Surprise*, in the LFR networks with 500 vertices, different mean degrees (*k*_m_), and different community-size heterogeneity. (a) *C*_max_=50 and (b) *C*_max_=100. Other parameters are the same as in Table S1.

**Figure S9.** Relative values of different quality functions for community partition identified by *Quasi-Surprise*, in the LFR networks with 1000 vertices, different mean degrees (*k*_m_), and different community-size heterogeneity. (a) *C*_max_=50 and (b) *C*_max_=100. Other parameters are the same as in Table S2.


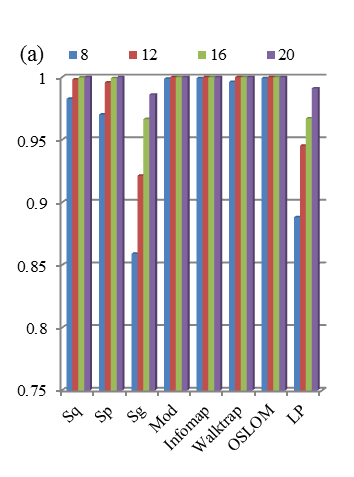

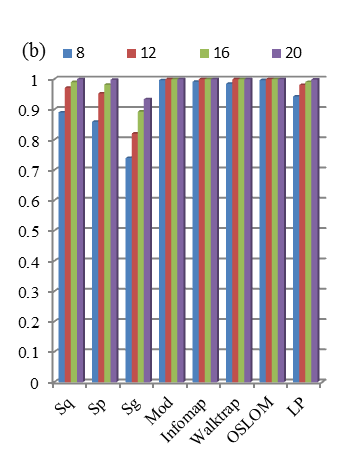


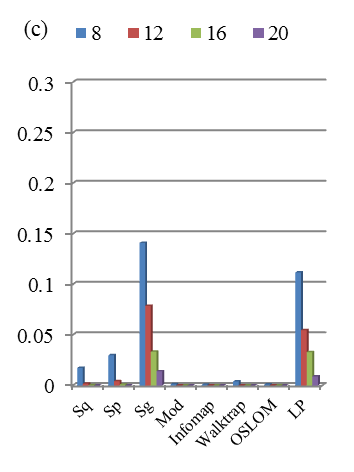

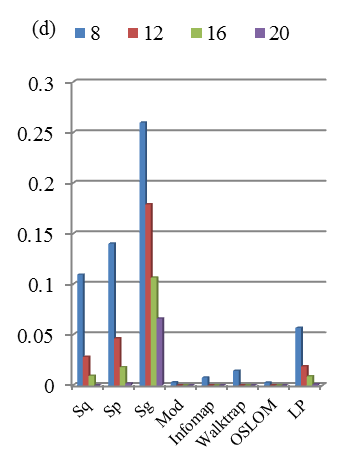


**Figure S10.** The normalized mutual information (NMI) of community structures identified by different methods, in the LFR networks with 500 vertices, different mean degrees (*k*_m_) and different community-size heterogeneity. Other parameters are the same as in Table S1. (a) *C*_max_=50 and (b) *C*_max_=100 for NMI. (c) *C*_max_=50 and (d) *C*_max_=100 for 1 minus NMI.


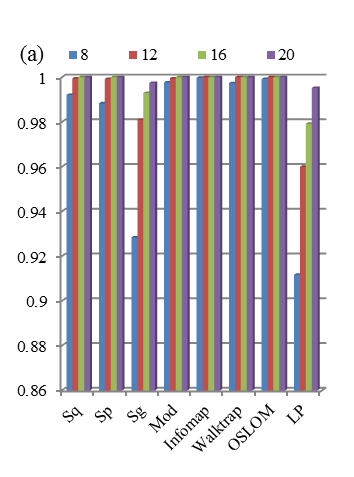

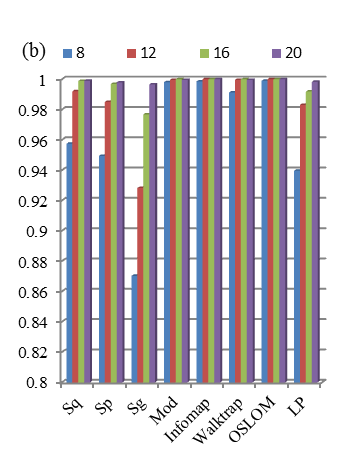


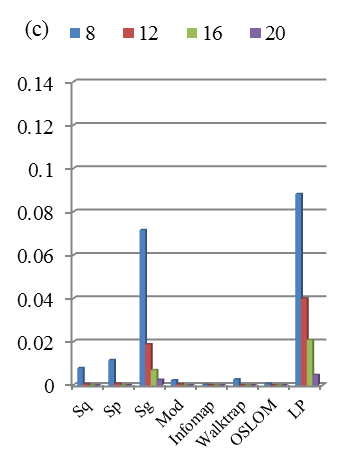

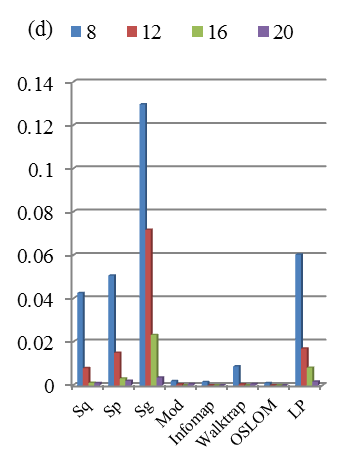


**Figure S11.** The normalized mutual information (NMI) of community structures identified by different methods, in the LFR networks with 1000 vertices, different mean degrees (*k*_m_) and different community-size heterogeneity. Other parameters are the same as in Table S2. (a) *C*_max_=50 and (b) *C*_max_=100 for NMI. (c) *C*_max_=50 and (d) *C*_max_=100 for 1 minus NMI.

## **6. Analysis of multi-scale methods**

Here, we further display the effectiveness of the multi-scale Significance as well as Modularity in detecting communities at different scales. The results show that they are able to identify the partitions of the pre-defined scales in the networks.

Figure S12 shows the results of multi-scale Significance in two kinds of multi-scale networks.

Figure S13 shows the results of multi-scale Modularity in two kinds of multi-scale networks.

The critical number of communities (dense subgraphs) in partition transition will be changed due to the introduction of the resolution parameter, after the single-scale methods are extend to multi-scale case. In the two-level networks, for example, the critical number of communities (dense subgraphs) can be estimated as $r_{2}^{\#}\text{=}\gamma\cdot r_{2}^{\text{*}}$ when $r_{2}^{\#}/\gamma$ is large. For Quasi-Surprise,

$r_{2}^{\#}\text{=}\gamma\cdot\frac{1-q_{2}}{q_{2}}\cdot\exp\{\frac{1}{\beta}D(q_{1}||q_{2})\}\cdot2^{\frac{q_{1}}{\beta}+1}$.

For Significance,

$r_{2}^{\#}=\gamma\cdot p'\cdot\exp\left(\frac{2H(p_{2})-H(p_{1})}{2p_{2}-p_{1}}\right)$.

For Modularity,

$r_{2}^{\#}=\gamma\cdot\frac{p_{in}+p_{out1}+p_{out2}}{p_{out1}}$.

With the decrease of the resolution parameter $\gamma$, the critical number $r_{2}^{\#}$ of communities (dense subgraphs) decreases, and thus the communities will tend to merge. With the increase of the resolution parameter $\gamma$, the critical number $r_{2}^{\#}$ of communities increases, and thus the communities will tend to split.

The multi-scale versions of the methods provide alternatives to analyze the structures at different scales. However, because of nonlinearity of Quasi-Surprise and Significance, the physical meaning of their resolution parameters is not as clear as that of Modularity. Therefore, the extension of the methods to multi-scale networks still deserves further study.

**Figure S12.** Community partitions identified by Significance in (A)-(B) two-level networks with *n*_c_=10 and *r*=20, and (C)-(D) hierarchical networks with 256 vertices and two scale community structures. Nc is the identified number of communities in the networks. NMI-1 denotes the NMI between identified and level-1 partitions. NMI-2 denotes the NMI between identified and level-2 partitions.

**Figure S13.** Community partitions identified by multi-scale Modularity in (A)-(B) two-level networks with *n*_c_=10 and *r*=20, and (C)-(D) hierarchical networks with 256 vertices and two scale community structures. Nc is the identified number of communities in the networks. NMI-1 denotes the NMI between identified and level-1 partitions. NMI-2 denotes the NMI between identified and level-2 partitions.

## **7. Real-world networks.**

Here, the above methods are applied to a set of real-word networks, including karate network , dolphin network , Les Miserables (Les Mis.), polbooks network, football network , Jazz , C. elegans neural network . It is not easy to directly compare the methods in the real-world networks, and Table S7 therefore shows the number of communities identified by different methods.

Similar to the results in the model networks, Quasi-Surprise also tends to generate more communities in the real-world networks than Modularity, because it has higher resolution than Modularity. Moreover, original Surprise as well as Significance can find more communities in the networks than other methods, because they have higher resolution than others.

**Table S7.** The number of communities in various real-world networks, identified by different methods.

| **Method** | ***Sq*** | ***Sp*** | ***Sg*** | ***Mod*** | ***Infomap*** | ***Infomod*** | ***Walktrap*** | ***OSLOM*** | ***LP*** |
| --- | --- | --- | --- | --- | --- | --- | --- | --- | --- |
| Karate | 6 | 14 | 15 | 4 | 3 | 2 | 6 | 2 | 11 |
| Dolphin | 17 | 22 | 22 | 5 | 6 | 2 | 5 | 2 | 10 |
| Les Mis. | 25 | 30 | 25 | 7 | 9 | 4 | 9 | 4 | 15 |
| Polbooks | 8 | 19 | 28 | 5 | 5 | 3 | 4 | 2 | 6 |
| Football | 12 | 15 | 15 | 9 | 12 | 10 | 10 | 11 | 11 |
| Jazz | 16 | 36 | 36 | 4 | 6 | 13 | 8 | 5 | 3 |
| C. elegans | 25 | 62 | 67 | 5 | 8 | 7 | 22 | 3 | 2 |

## **References**
